# Are Machine Learning methods effective in detecting undiagnosed atrial fibrillation in primary care settings using electronic health records? A systematic review

**DOI:** 10.1371/journal.pdig.0001009

**Published:** 2025-10-14

**Authors:** Mhd Diaa Chalati, Chetan Shirvankar, Genevieve Gore, Abhinav Sharma, Samira Abbasgholizadeh-Rahimi

**Affiliations:** 1 Department of Family Medicine, McGill University, Montreal, Quebec, Canada; 2 Mila-Quebec AI Institute, Montreal, Quebec, Canada; 3 Faculty of Dental Medicine and Oral Health Sciences, McGill University, Montreal, Quebec, Canada; 4 Schulich Library of Physical Sciences, Life Sciences, and Engineering, McGill University, Montreal, Quebec, Canada; 5 Research Institute of the McGill University Health Centre, McGill University, Montreal, Quebec, Canada; 6 Lady Davis Institute for Medical Research, Jewish General Hospital, Montreal, Quebec, Canada; Liverpool John Moores University - City Campus: Liverpool John Moores University, UNITED KINGDOM OF GREAT BRITAIN AND NORTHERN IRELAND

## Abstract

Atrial fibrillation (AF) increases the risk of stroke, heart failure and mortality. Current screening guidelines fail to detect AF effectively, and existing models have limited applicability in primary care. Electronic health records (EHRs) provide an opportunity to apply machine learning (ML) for automated AF detection; however, their performance relative to standard care remains unclear. We conducted a systematic review to evaluate the effectiveness, quality, and applicability of EHR-based ML models for detecting AF in primary care. The review is informed by Joanna Briggs Institute and Preferred Reporting Items for Systematic reviews and Meta-Analyses (PRISMA) guidelines. We searched seven databases from inception to May 2023. Eligible studies involved adults in primary care where ML models using EHRs were compared to standard care. The primary outcome was the detection of undiagnosed AF; secondary outcomes examined impacts on patients, healthcare providers, and systems. Data were extracted using CHARMS, risk of bias and applicability were evaluated through PROBAST and MI-CLAIM checklists. This review was registered in International Prospective Register of Systematic Reviews (CRD42023390603). From 4,536 references screened, 16 studies were included. Among these, 14 (87%) were retrospective cohort studies, one (6%) was prospective, and one (6%) was a randomized controlled trial. Random forest classifiers were the most common ML model (7 studies, 43%). Only 4 studies (25%) underwent external validation, and 8 (53%) were at high risk of bias. Model discrimination (AUROC) ranged from 0.71 to 0.948, with 8 (50%) outperforming controls. Combining ML with clinical tools (3 studies, 19%) significantly improved discrimination compared to ML models alone. Reviewed models identified gout as a nontraditional predictor of AF and demonstrated that dynamic measures of BMI, blood pressure, and heart failure diagnosis were stronger predictors than static measures. EHR-based ML models show promise for improving AF detection in primary care compared to standard care. Their clinical applicability, however, is limited by insufficient external validation, high risk of bias, and variable performance. Future research should prioritize external validation, evaluation in clinical trials and the integration of predictors routinely available in primary care.

## Author summary

Atrial fibrillation is a common condition that significantly increases the risk of serious health problems such as strokes and heart failure. Despite its impact, it often goes undiagnosed in its early stages due to the lack of reliable clinical tools, leading to thousands of preventable hospitalizations each year. While machine learning has shown potential in improving detection, much of the research has focused on models using electrocardiogram data. In contrast, our review emphasizes the use of electronic health records, a widely available yet underutilized resource in primary care, for automated risk assessment. In this review, we examined how machine learning models based on electronic health records could improve the detection of undiagnosed atrial fibrillation. By using routinely collected health information, we showed that these tools could identify patients at risk earlier and more accurately, enabling timely interventions that improve health outcomes. However, their widespread use is limited by challenges such as inconsistent performance, insufficient testing in diverse real-world settings, and biases in data. Addressing these limitations is crucial to realizing the full potential of this approach. Our review advances the field by synthesizing evidence, identifying critical gaps, and providing a roadmap for future research. By emphasizing robust testing, active collaboration with healthcare providers and patients, and tailoring these tools to primary care needs, our work lays the foundation for making machine learning a trusted and practical solution for early detection of atrial fibrillation.

## Introduction

Atrial fibrillation (AF) is the most prevalent arrhythmia encountered in family medicine, with estimates from the Canadian Cardiovascular Society indicating that up to a million Canadians experience either silent or paroxysmal AF [1]. Left untreated, AF substantially increases the risk of severe stroke—by three to five times [2], elevates mortality rates even after adjusting for other cardiovascular factors [3] and increases the risk of heart failure [4–6].

Despite the high prevalence and significant risks associated with AF, current primary care screening methods remain inadequate globally. For instance, in Canada, no unified guideline for AF screening exists, with the Canadian Cardiovascular Society’s recommendation limited to opportunistic screening for patients aged 65 and older, involving pulse palpation during annual check-ups [1]. Opportunistic screening often fails to detect silent AF due to its lack of systematic approach.

Traditionally, patients were flagged for AF screening based on age alone, with one study showing a number needed to treat (NNT) of 111 [7]. Himmelreich et al.‘s systematic review also identified the Cohorts for Heart and Aging Research in Genomic Epidemiology for Atrial Fibrillation (CHARGE-AF) model as the most successful for predicting incident AF over a five-year window. Despite widespread validation and readily available clinical metrics, CHARGE-AF has limitations such as primarily studied in three American cohorts which is limiting its applicability to non-white populations. Besides, it relies on static numerical values that require significant manual input from primary care physicians, adding to their workload. It also falls short in primary care when it comes to systematic and effective detection [8]. Consequently, it is estimated that 20% of AF cases are only diagnosed after a patient experiences their first stroke [9] leading to thousands of hospitalizations per year [10].

Given the high risks associated with untreated AF and the limitations of current screening methods, a more reliable, efficient, and integrated approach is urgently needed in primary care. Machine learning (ML) offers a promising solution by analyzing large volumes of patient data to identify AF risk patterns with greater accuracy. While wearable technologies are gaining greater acceptance in cardiovascular disease states such as heart failure [11,12], ML applications in AF detection are already showing potential. Notably wearables integrated with convolutional neural networks (CNNs) have demonstrated potential for AF monitoring [13], as well as for improving AF detection from ECGs [14]. However, these models often rely heavily on ECG-derived data and require active provider input, which limits their scalability in primary care.

This has led to a growing interest in using ML with electronic health records (EHRs), which contain readily available patient information crucial for AF risk assessment. EHR-based ML models could analyze comprehensive data, including demographics, comorbidities, and health history, to provide an automated and effective screening tool. While evidence on this approach is expanding, it remains unclear how these EHR-based models perform compared to standard care. Many studies lack direct comparisons, and questions remain about the generalizability of ML models in primary care due to variability in validation methods and dataset limitations. A systematic synthesis of current evidence is therefore necessary to assess the benefits and limitations of ML-based tools for AF screening using EHR.

The primary aim of this systematic review is to evaluate the effectiveness of ML models in detecting undiagnosed AF in primary care settings using EHR data compared to standard screening practices. Additionally, this review seeks to assess the quality of these models by examining their validation methods and risk of bias, and to explore the clinical relevance of ML models by analyzing their impact on patients, healthcare providers, and healthcare systems. This will provide a comprehensive evaluation of ML-based AF detection tools using EHR, supporting primary care providers in making informed decisions about integrating these technologies into routine practice. Enhanced EHR-based ML screening could enable early AF detection, reducing strokes and hospitalizations. For clarity, key machine learning terms used this review are defined in Table 1.

**Table 1:** Glossary of machine learning terms used in this review.

Machine learning is a method in which algorithms learn patterns from data to make predictions or decisions without explicit task-specific programming.
Supervised learning: ML approach where models are trained on labeled data (i.e., data with known outcomes).
Unsupervised learning: ML approach where models are trained on unlabeled data to discover hidden patterns or clusters.
Random forest classifier (RFC): An algorithm that makes predictions by combining the results of many decision trees.
Neural networks (NN): A ML model made of layers of interconnected nodes (mimicking neurons) that can learn complex, non-linear relationships in data.
Natural language processing (NLP): Computational methods that process and extract information from unstructured text (natural language), such as doctors’ notes in electronic health records (EHRs).

## Methods

The protocol for this systematic review was informed by the Joanna-Briggs Institute (JBI) guidelines [15] and registered on the International Prospective Register of Systematic Reviews (PROSPERO; ID: CRD42023390603) found in S1 Protocol. The PICOS (population, intervention, comparator, outcome) framework [16] was used to develop the inclusion and exclusion criteria for suitable studies. This systematic review followed the Preferred Reporting Items for Systematic Reviews and Meta-Analyses (PRISMA) checklist [17].

### Search strategy

An information specialist/librarian (GC) developed the search strategy and conducted a comprehensive search of electronic databases from the date of inception to May 2023. The search strategy was applied to Ovid-MEDLINE and translated to other databases (Ovid-MEDLINE, Embase, CINAHL, Cochrane CENTRAL, Web of Science, IEEE Xplore, Scopus) after validation and revision with the team. The relevant search strategy can be found in S1 Appendix.

### Eligibility criteria

We included peer-reviewed publications in English using the PICOS framework. *Population*: Studies involving adult populations (aged >18 years) were included. *Intervention*: Oly studies that developed and/or applied ML methods using data from EHRs or primary care databases to detect new cases of AF within a primary care context were included. Accepted ML methods included algorithms such as decision trees, neural networks, support vector machines, Bayesian networks, and other models specifically designed for AF detection. Studies that utilized ML models developed exclusively with variables from ECGs, imaging data, or wearable devices without being integrated into EHR were excluded. However, studies that incorporated these models as clinical variables within EHR-based ML models or used them as part of a combined model with validated clinical risk scores for application in a primary care setting, were included. *Comparison*: Standard care practices or validated clinical risk scores for AF.

Outcome: The primary outcome of interest was the incidence of new AF diagnoses following the application of ML methods. Secondary outcomes included those related to patients (e.g., clinical outcomes, functional improvements, quality of life, patient satisfaction, and patient knowledge), healthcare providers (e.g., performance measures, provider satisfaction, skill enhancement, and patient-provider relationship quality), and healthcare systems (e.g., access to care, cost-efficiency, safety metrics, equity, healthcare utilization, and population health indicators). *Settings:* Eligible studies were conducted within primary care or outpatient settings where patients were managed by healthcare providers, such as general practitioners, family physicians, nurse practitioners, physician assistants, general internists, and geriatric primary care providers. Studies focused solely on pediatric populations, emergency rooms, or inpatient settings or those without a clear focus on primary care were excluded.

### Articles screening and selection process

Two independent reviewers (DC & CS) screened all the articles’ titles and abstracts using the predetermined eligibility criteria. After excluding irrelevant articles and duplicates, selected articles were moved to full-text review to further assess their relevance. Reviewers conducted their assessments independently, with decisions blinded from one another. Any disagreements were resolved by consulting a third reviewer (SAR). Covidence software was used to facilitate the screening and selection process.

### Data extraction

We used the Checklist for Critical Appraisal and Data Extraction for Systematic Reviews of Prediction Modeling Studies (CHARMS) for data extraction (Moons et al., 2014). The CHARMS checklist is structured to systematically extract data from primary studies reporting on prediction models as well as to assess potential sources of bias in ML method selection. Key categories extracted included study information (e.g., data source), participant details (e.g., population characteristics), outcomes (e.g., outcome type and blinding of assessment), candidate predictors (e.g., predictor type), model development (e.g., sample size, handling of missing data, and predictor selection), model performance (e.g., calibration, classification measures), and model evaluation (e.g., internal vs. external validation).

In addition to these criteria, we captured further details on study design, such as the clinical question, ML method, control groups, intended use of results, validation processes, and clinical outcomes (e.g., patient-related, provider-related, and healthcare system-related). For each study, we also documented the final variables used to develop the ML models.

We customized and tested the data extraction framework on several initial articles to ensure comprehensiveness and consistency. We specifically reported on the highest-performing AI systems in AF detection, whether their performance was achieved by an ML model alone or in combination with other tools, and compared it against control groups. In this context, we identified model discrimination—the ability to accurately distinguish between individuals with and without AF—as a key metric to measure the performance of ML models. Significance was defined as by a P value < 0.05 or non-overlapping confidence intervals as reported in each article.

### Quality assessment

We used the Prediction Model Risk of Bias Assessment Tool (PROBAST) for quality assessment [18]. Data extracted via CHARMS was appraised with PROBAST, which evaluates risk of bias and applicability in prognostic model studies across four domains: participants (e.g., selection bias), model predictors (e.g., bias from inconsistencies in predictor definitions and selection), outcome (e.g., bias due to outcome misclassification), and analysis (e.g., bias from inappropriate statistical methods in model development). To streamline this process, we implemented the ready-to-use template by Fernandez-Felix et al., 2023 [19]. Additionally, we applied the Consolidated Standards of Reporting Trials–Artificial Intelligence (CONSORT-AI) guidelines to assess the quality of clinical trials involving AI, ensuring comprehensive adherence to reporting standards [20].

### Applicability

Alongside the applicability assessment section in PROBAST, we used the Minimum Information about Clinical Artificial Intelligence Modeling (MI-CLAIM) checklist [21] to evaluate the clinical relevance of ML models. MI-CLAIM facilitates the evaluation of the clinical impact and the reproducibility of AI models in clinical AI studies and includes six key areas: study design, data partitioning for model training and testing, data optimization, performance evaluation, model examination, and code sharing. This comprehensive approach ensured the systematic extraction of relevant data for our review. Integrating MI-CLAIM into the data extraction process enabled us to address critical aspects of validity, fairness, and replicability, contributing to a robust and nuanced evaluation of AI’s role in AF screening.

### Data synthesis and analysis

Given the heterogeneity among studies and the diversity in performance metrics reported, a meta-analysis was not feasible; instead, we conducted a narrative and descriptive synthesis. This involved using textual descriptions of each study, grouping and clustering by relevant characteristics, and tabulating key effectiveness measures, with a particular focus on model performance metrics such as sensitivity, specificity, positive predictive value, accuracy, ROC-AUC, PRC-AUC, and F1 score. We also explored sources of variation across studies, including differences in study design, methodology, populations, interventions, control groups, and outcomes. Bias assessment was reported in accordance with PROBAST, utilizing the template developed by Fernandez-Felix et al., 2023 [19].

## Results

### Study selection

**Fig 1:**
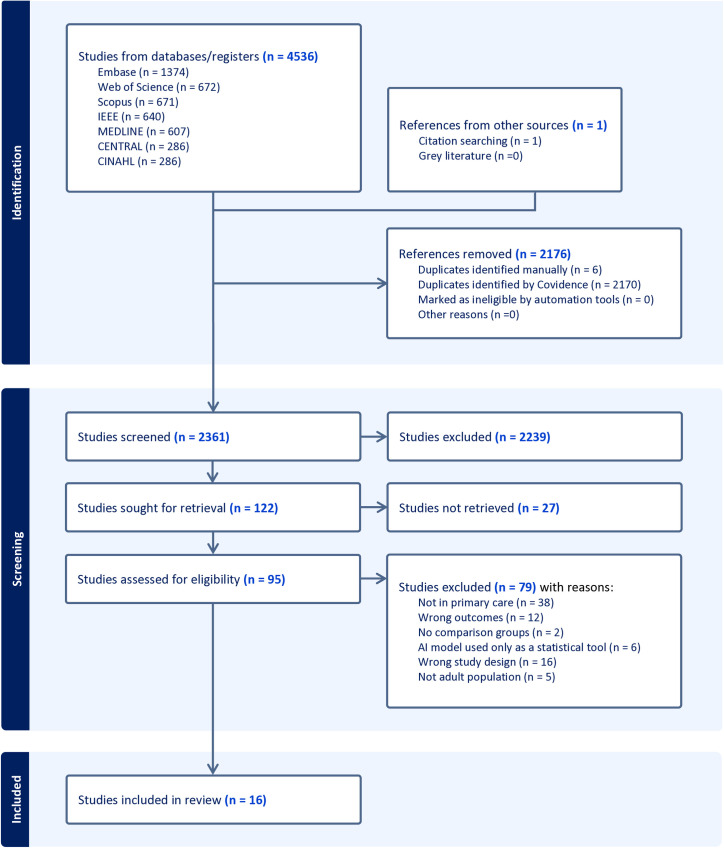
PRISMA flow diagram. Summary of study selection process for inclusion in the systematic review. Abbreviations: PRISMA, preferred reporting items for systematic reviews and meta-analyses.

Fig 1 shows the PRISMA flow diagram for the study selection process in this review. Our initial search across seven databases yielded 4,536 references, with one additional study manually included through citation tracking due to its relevance to our inclusion criteria. After removing duplicates, 2361 unique records remained for title and abstract screening. Following the first round of screening, 122 studies were selected for full-text review. Ultimately, 16 articles met the eligibility criteria and were included for data extraction. Fig 1 provides a breakdown of exclusion reasons. S1 Checklist shows PRISMA checklist.

### Study characteristics

**Fig 2:**
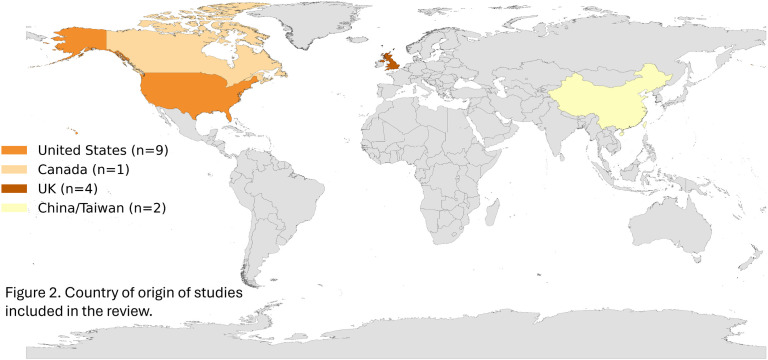
Geographic distribution of included studies. Map displaying the countries where included studies were conducted. Source shapefile: Map created in Python using shapefile data from the geo-countries dataset, which is derived from Natural Earth and licensed under the Open Data Commons Public Domain Dedication and License (PDDL).

*Studies location/context*: Of the 16 articles, the majority were conducted in the United States (n = 9), followed by the United Kingdom (n = 4), Canada (n = 1), and China/Taiwan (n = 2). Twelve studies developed ML methods using outpatient databases, while four studies [22–25] utilized databases integrating both inpatient and outpatient data. Most articles (n = 15, 94%) were published between 2019 and 2022. A visual map of study locations is provided in Fig 2. Table 2 provides an overview of the characteristics of studies included in this review including detailed comparisons of datasets and model characteristics.

**Table 2:** Studies Characteristics.

First author	Clinical setting	Country	Study design	Dataset used for ML development	AI type	Control Group	Outcome
Elkin 2021 [26]	Primary care	New York, USA	case-control study	Allscripts outpatient electronic records at the University at Buffalo’s (UBMD)	Semi-supervised high definition-NLP	Clinicians’ assessments. VASCHADS	NVAF diagnosis
Shah 2020 [22]	Inpatient and outpatient	Utah, USA	Retrospective Cohort	EDW from University of Utah Health	Supervised rules-based method for NLP approach (pyConText algorithm)	Combined LR model + ICD billing codes for AFib	Presence of AF
Karnik 2012 [27]	Primary care	Marshfield Clinic, USA	Retrospective Cohort	Marshfield Clinic electronic health records	Supervised ML methods (naïve Bayes, SVM, LR, RF)	LR	AF incidence
Hill 2019 [28]	Primary care	UK	Retrospective Cohort	CPRD	Supervised ML methods (neural network, LASSO, RF, SVM)	CHARGE-AF, LR	AF prevalence
Tiwari 2020 [23]	Inpatient and outpatient	Colorado, USA	Retrospective Cohort	UCHealth hospital	Supervised ML methods (Naive Bayers, LR, RF, neural network (Deep, Shallow)	LR model with standard risk factors	AF incidence
Nadarajah 2023 [29]	Primary care	Leeds, UK	Retrospective Cohort	CPRD-GOLD dataset.	Supervised RFC	CHA2 DS2 -VASc, C2HEST	AF incidence at 6 months
Shah 2020 [25]	Inpatient and outpatient	Utah, USA	Retrospective Cohort	Internal validation: EDW from University of Utah Health. External validation: NMH in Chicago	Supervised ML methods for NLP	Reference standard: clinician review of charts	AF prevalence
Christopoulos 2020 [30]	Primary care	Ohio, USA	Retrospective Cohort	MCSA	AI-ECG	CHARGE-AF score	AF incidence
Sekelj 2021 [31]	Primary care	London, UK	Retrospective Cohort	WSIC	Time-varying Neural Networks	No control group	New AF
Khurshid 2022 [32]	Primary care	Massachusetts, USA	Retrospective Cohort	C3PO	AI-ECG	CHARGE-AF score	5 years Incident AF/Aflutter
Ashburner 2022 [33]	Primary care	Massachusetts, USA	Retrospective Cohort	Internal validation: Primary Care Practice-Based Research Network at MGH. External validation: Brigham and Women’s Hospital with primary care visits	Unsupervised ML methods for NLP	CHARGE-AF	5 years Incident AF
Bhattacharya 2021 [34]	Outpatient	Baltimore, USA	Retrospective Cohort	-HCM Registry	Supervised ML methods (naïve Bayes, LR, decision tree, and RF)	CHARGE-AF, LR	New AF
Sung 2022 [24]	Inpatient and outpatient	Taiwan, China	Retrospective Cohort	DRD stroke registry	Supervised ML methods for NLP (XGBoost) Model A: structured. Model B: unstructured. Model C: combined A + B	Control: CHASE-LESS.	new AF poststroke in 10.2 month follow up
Dykstra 2022 [35]	Outpatient	Calgary, Canada	Prospective Cohort	CIROC	Supervised ML methods (RSV)	CHARGE-AF score, CHEST	new onset AF over 4-years
Hill 2022 [36]	Primary care	England, UK	prospective, randomized controlled trial	National Institute for Health Research Clinical Research Network	Time-varying Neural Networks	Routine care only	Proportion of AF diagnoses and related arrhythmias
Hu 2019 [37]	Primary care	Taiwan, China	Retrospective Cohort	Internal validation cohort: LHID. External validation cohort: NHIRD inpatients datasets	Supervised ML methods (RFC)	CCI score	New AF

Abbreviations: ML, machine learning; NLP, natural language processing; SVM, support vector machines; LR, logistic regression; RF, random forest; RFC, random forest classifier; RSV, random survival forest; AI-ECG, convolutional neural network–enabled electrocardiography; NVAF, non-valvular atrial fibrillation; CPRD, the UK Clinical Practice Research Datalink; MCSA, mayo clinic study of aging; WSIC dataset, whole systems integrated care; [JHH]-HCM, Johns Hopkins hospital -hypertrophic cardiomyopathy registry; C3PO, community care cohort project dataset; CIROC, cardiovascular imaging registry of calgary; EDW, enterprise data warehouse; NMH, Northwestern memorial healthcare; LASSO (), CHARGE-AF (), CCI, charlson comorbidity index; DRD stroke registry, ditmanson research database; LHID, longitudinal health insurance database; NHIRD, national health insurance research database; USA, United States of America; UK, United Kingdom; MGH, Massachusetts general hospital.

**Fig 3:**
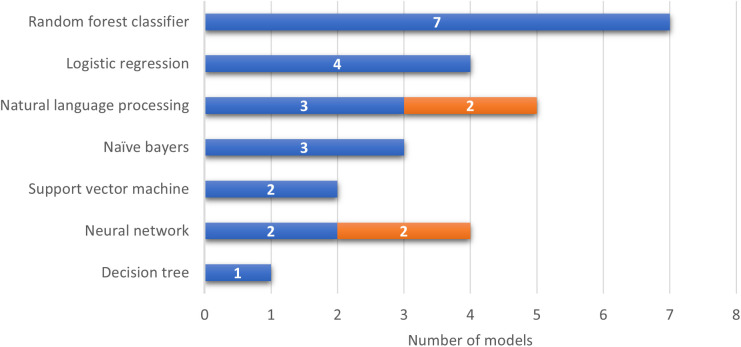
Distribution of machine learning models across included articles. Bar graph showing the frequency of different ML model types used among the included studies. Abbreviations: ML, machine learning; RF, random forest; SVM, support vector machine; LR, logistic regression; ANN, artificial neural network.

*Data source*: Among the 16 articles reviewed, 12 (92%) developed models exclusively using readily available EHR data. The rest of the studies included additional data modalities integrated with EHR data. For example, Christopoulos et al [30] developed a model that combined ECG data with EHR-based tools, which was later validated and tested on a larger sample by Khurshid et al. [32]. Similarly, two studies [34,35] incorporated imaging data alongside EHR data for their model development. Although these additional data sources were utilized, they were integrated with EHR data, meeting the inclusion criteria for this review. *Study Designs*: Most articles (n = 14, 87%) adopted retrospective cohort study designs. The remaining studies included a prospective cohort study design [35], and a prospective randomized clinical trial [36]. *Intervention (ML Models characteristics):* Among the 16 articles reviewed, a total of 13 unique models were identified. Notably, one model was externally validated and later tested in a clinical trial, as reported across two separate articles, and two studies [30,32] utilized the same model. Of these 13 models, the majority were supervised models (n = 11, 85%) and primarily relied on structured data (n = 13, 100%). Random forest classifiers were the most commonly used algorithm (n = 7, 54%). Additionally, deep learning models (n = 4, 31%) were employed, including time-varying neural networks [28], single-layer shallow neural networks [23], and convolutional neural networks (CNNs). The CNN-based AI-ECG model, originally developed by Attia et al. [38], was later implemented by Khurshid et al [32] and Christopoulos et al [30]. Fig 3 illustrates the ML models evaluated in each article (not the final model). *Clinical comparator:* The most frequently used control for comparison was the CHARGE-AF score (n = 6, 38%) followed by logistic regression models (n = 3, 19%) and clinician assessment (n = 3, 19%). Other control scores included C2HEST/CHADS, AS5F/CHseless, and the Charlson Comorbidity Index.

### Taxonomy

We propose a taxonomy (Table 3) to categorize the models based on three key dimensions: ML type, validation approach, and data type. Rather than listing all models tested in each study, we included only the best-performing machine learning models—based on discrimination performance. The taxonomy highlights the frequent use of random forest models, the limited use of external validation, and the inclusion of imaging or ECG data input alongside structured EHRs in a few models.

**Table 3:** A taxonomy of the types of ML models highlighting the best performing AI model within each study.

ML Type	Validation	Data Type	Studies
**Traditional ML**			
RF	Internal only	Structured EHR	Karnik 2012 [27], Hu 2019 [37], Nadarajah 2023 [29]
LR + NB	Internal only	Structured EHR + Imaging	Bhattacharya 2021 [34]
LR + TFIDM	External	Structured EHR + NLP	Shah 2020 [25]
**Survival ML**			
RSF	Prospective	Structured EHR + Imaging	Dykstra 2022 [35]
**NLP**			
NLP – Rules-Based	Internal only	Free Text	Shah 2020 [22]
**Deep Learning**			
NN	Internal only	Structured EHR + Imaging	Hill 2019 [28]
NN	Prospective (RCT)	Structured EHR + Imaging	Hill 2022 [36]
NN	External	Structured EHR	Sekelj 2021 [31]
Shallow NN	Internal only	Structured EHR	Tiwari 2020 [23]
CNN-based AI-ECG	Internal only	Structured EHR + ECG	Christopoulos 2020 [30]
CNN-based AI-ECG	External	Structured EHR + ECG	Khurshid 2022 [32]
**Hybrid**			
NLP + ML(un-supervised)	External	Structured EHR + NLP	Ashburner 2022 [33]
ML + NLP – Semi-supervised	Internal only	Free Text	Elkin 2021 [26]
Supervised ML methods for NLP (Boosted Trees (XGBoost))	Internal only	Structured EHR + NLP	Sung 2022 [24]

Abbreviation: LR + NB (Logistic regression and naïve Bayes classifiers (HCM-AF-Risk Model)), RF (Random Forest), LR (Logistic regression), TFIDM (term frequency-inverse document frequency method), RSF (Random survival forest), CNN (convolutional neural network), NLP (Natural language processing)

### Outcomes

Study outcomes primarily reported AF incidence or prevalence over periods ranging from 6 months to 5 years. Table 4 categorizes outcomes into patient-related, provider-related, and healthcare system-related outcomes.

**Table 4:** Performance metrics of AI systems compared to controls and clinical outcomes.

First author (year)	Performance metrics	Best performing AI system (AI combined with other clinical tools)	AI system performance compared to control group: see Table 2	Clinical outcome		
				Patient-related	Provider-related	System-related
Elkin 2021 [26]	AUC, Sensitivity, Specificity, PPV, NPV, F score	Combined machine learning methods (semi supervised and supervised methods) for natural language processing	**Internal validation:** AUC combined NLP+VASC = 0.914 (95% CI 0.896-0.933) vs structured data 0.863 (CI 0.838-0.887). Sensitivity OR = 1 vs 0.54 P < .001. PPV OR = 0.93 vs 0.95 P = 0.24. F score = 0.964 vs 0.686	Improved detection and potentially preventing 176,537 strokes	Enhanced sensitivity for CHA2DS2-VASc score.	Reduced cost by potentially saving US $13.5 billion if ML applied in USA.
Shah 2020 [22]	Sensitivity, Specificity, FN, FP, F score, AUC	Comprehensive: combined logistic regression models, Billing codes and NLP	**Development model: comprehensive vs NLP vs control:** AUC = 0.887 vs 0.801 vs 0.798. Sensitivity = 0.903 vs 0.972 vs 0.785. Specificity = 0.870 vs 0.630 vs 0.812 Comprehensive vs NLP P < 0.05. NLP vs control P = 0.91	Improved detection compared to control.	High diagnosis accuracy compared to NLP or control alone	Not specified
Karnik 2012 [27]	Precision, Recall, F measure	On numeric data: Random Forest classifier utilizing Textual dataset with TF-IDF encoding (MTD2) all-year window.	**Internal validation:** Precision = 58% vs 56.7%. Recall = 61.7 vs 59.4. F score = 60.1 vs 57.6	Modest performance compared to logistic regression.	Not specified	Not specified
Hill 2019 [28]	Specificity, PPV, NNS, AUROC	Time-varying Neural Networks	**Internal validation: ML vs control vs LR:** (relative change vs LR) Specificity = 74.9% vs 61.0% vs 52.0%. PPV = 11.5% vs 7.9% vs 6.5%. NNS = 9 vs 13 vs 15. AUROC = 0.827 (19%) vs 0.725 (4.3%) vs 0.695	Earlier diagnosis and Identification of new risk factors for AF	Improved precision in the model’s performance compared to control	Enhanced screening efficiency with NNS, reduced from 13 to 9.
Tiwari 2020 [23]	F1 Score, AUC, Training time	Single layer shallow neural network	**Internal validation: ML vs LR** AUC = 0.800 vs 0.794, F1 Score = 0.110 vs 0.079.	Similar performance compared to control.	Not specified	Potentially enhanced scalability by applying ML to EHR participating in OMOP
Nadarajah 2023 [29]	AUROC, sensitivity, specificity, PPV,NPV, Brier Score, Calibration Slope	Random Forrest Classifier	**Internal validation:** AUROC = 0.824 (95% CI 0.814 to 0.834) vs 0.784 (95% CI 0.773 to 0.794) Sensitivity = 0.781. Specificity = 0.731. PPV = 2.5% NPV = 99.8% Calibration Slope = 0.782. Brier Score = 0.069.	Improved detection of high-risk patients compared to control.	Robust model performance across different sex and ethnic groups.	Not specified
Shah 2020 [25]	Accuracy, PPV, NPV, Sensitivity, Specificity, F-Score, AUC	Logistic regression, using 1000 features, 100 stop words, and term frequency-inverse document frequency method	**Internal validation:** Accuracy = 0.916. AUC = 0.91. F-Score = 0.943. PPV = 0.961. NPV = 0.797. Sensitivity = 0.925. Specificity = 0.887 **External validation:** Accuracy = 0.870. AUC = 0.80. F-Score = 0.922 PPV = 0.951. NPV = 0.519. Sensitivity = 0.895. Specificity = 0.711. AUC, F-score of int vs ext validation P < 0.001	Not specified	Improved detection using free text analysis rather than structured ICD or billing codes.	not specified
Christopoulos 2020 [30]	C-statistic	Statistical association (AI-ECG+ CHARGE-AF score)	**Internal validation:** C-index for (AI-ECG+ CHARGE-AF) vs AI-ECG vs CHARGE-AF = 0.72 (95% CI, 0.69–0.75) vs 0.62 (95% CI, 0.66–0.72) vs 0.69 (95% CI, 0.66–0.71)	Mild improvement in detection when combining AI-ECG with CHARGE-AF	Persistent performance of AI-ECG after 8 years compared to CHARGE-AF.	not specified
Sekelj 2021 [31]	Sensitivity, Specificity, PPV, NPV, Potential NNS, AUROC	Time-varying Neural Networks	**External validation: At 75% sensitivity:** Specificity = 82%, PPV = 11.3%, NPV = 99.1%, NNS = 9 AUROC = 0.87. In patients >65: AUC was 0.71	Decreased detection performance among patients >65 years old.	Not specified.	Not specified.
Khurshid 2022 [32]	AUROC, Precision, Time dependent metrics, HR, calibration slope	Statistical association (AI-ECG + CHARGE-AF score)	**Internal validation at 5 years: Combined ML vs ML vs CHARGE-AF** AUROC = 0.838 vs 0.823 vs 0.802, Precision = 0.30 vs 0.21 P < 0.05 (Combined ML vs CHARGE-AF P < 0.05) **External validation at 5 years in the BMH:** AUROC = 0.777 vs 0.743 vs 0.752. Precision = 0.21 vs 0.17 P < 0.05 (Combined ML vs CHARGE-AF P < 0.05) **External validation at 2 years in the UK Biobank:** AUROC = 0.746 vs 0.732 P = 0.28. Precision = 0.059 vs 0.02 P < 0.05	Improved detection across internal and externally validated combined CH-AI model	Not specified.	Potential application in wearable devices given stable performance in a single ECG lead.
Ashburner 2022 [33]	C-statistic, NRI, calibration slope	Unsupervised learning methods for natural language processing + Codified EHR data	**Internal validation at 5 year follow up: Combined ML vs control** C-index = 0.735 vs 0.717 P = 0.002 Overall NRI compared to control = 0.070 (0.033–0.113) **External validation at 5 year follow up:** C-index = 0.750 vs 0.735 P = 0.001	Modest improvement in detection by incorporating narrative data from EHR using NLP compared to using codified data alone.	Not specified	Not specified
Bhattacharya 2021 [34]	C-index, specificity, sensitivity	Logistic regression and naïve Bayes classifiers (HCM-AF-Risk Model)	**Internal validation:** ML vs CHARGE-AF C-index = 0.80 vs 0.61 (P < 0.001). Specificity = 0.70 vs 0.60 (P < 0.001), Sensitivity = 0.74 vs 0.54 (P < 0.001). ML vs LR: C-index = 0.80 vs 0.79	Improved detection among HCM patients compared to clinical scores	Not specified	Not specified
Sung 2022 [24]	C-index, HR	Model C based on both structured data and unstructured textual data	**Internal validation:** C-index of Control vs Model A vs Model B vs Model C: 0.768 vs 0.791 vs 0.738 vs 0.840 Model A to control (p = 0.487). Model C to model B (p < 0.001) Model C to control (p = 0.005). Model B to control (p = 0.282)	Improved detection compared to traditional risk scores.	Not specified	Not specified
Dykstra 2022 [35]	C-index, time-sensitive AUC, NND	Random survival forest methods using all 115 variables and 20 top performing variables	**Internal validation:** Mean C-index: CIROC-AF-20 = 0.78 + /- 0.01 vs CHARGE-AF = 0.71 + /- 0.02 AUCs at 1-, 2- and 3-years: CIROC-AF-20 = 0.80, 0.79, and 0.78 vs CHARGE-AF = 0.72, 0.71 and 0.70 NND 3rd year follow up: CIROC-AF-20 = 2.18 + /- 0.14 vs CHARGE-AF = 2.96 + /- 0.41	Improved detection compared to historic AF scores.	Improved accuracy and stable performance over time in model using cardiac MRI data and EHR clinical predictors.	Improved NND compared to historic AF scores
Hill 2022 [36]	OR, NNS	Time-varying Neural Networks	**Odds Ratio: intervention vs Control:** Full analysis population 1.07 P = 0.625 High-risk population 1.15 P = 0.486 Research clinic population 1.31 P = 0.003 Per-protocol population 3.07 P = 0.001 NNS = 12	Improved detection of high risk patients especially when combined with ECG/Holter for 2 weeks.	Not specified	Enhanced screening by narrowing the target population.
Hu 2019 [37]	F score, AUC, recall, precision	Random forest classifier using Gini impurity	**Internal validation:** AUC = 0.948 [95% (CI) 0.947–0.949) vs 0.671 (95% CI 0.666–0.676). F1 score = 0.968. Precision score = 0.958. Recall score = 0.979 **External validation (on inpatient database):** AUC = 0.850 (95% CI 0.849–0.851) vs 0.747 (95% CI 0.663–0.832) F1 score = 0.983. Precision score = 0.977. Recall score = 0.988	Improved detection among Chinese individuals compared to CCI score	Not specified	Not specified

Abbreviations: AUC, area-under-curve; PPV, positive predictive value; NPV, negative predictive value; OMOP, observational medical outcomes partnership; NRI, net reclassification improvement; ML, machine learning; NLP, natural language processing; OR, odds ratio; FN, false negative; FP, false positive; MTD2, master text dataset; NNS, number needed to screen; AUROC, area under the receiver operating characteristic; LR, logistic regression; EHR, electronic health record; CHARGE-AF (); HR, Hazard ration; HCM, hypertrophic cardiomyopathy; NND, number needed to diagnose; ECG, electrocardiogram.

***Patient-Related Outcomes*:** Most studies (n = 11, 69%) reported improved clinical outcomes, including early diagnosis and identification of high-risk individuals, along with the identification of new predictors and risk factors. Other patient-related outcomes were not addressed. ****Provider-Related Outcomes****: Improvements in provider performance measures, particularly diagnostic accuracy, sensitivity, and precision, were reported in four studies. Additional provider-related outcomes included the persistence of model performance over time (n = 2), robust performance across different genders and ethnicities (n = 1), and enhanced detection using free-text data (n = 1). ****System-Related Outcomes****: Enhanced healthcare utilization was noted in three studies, as evidenced by improved numbers needed to screen (NNS) or detect (NND), leading to more efficient AF screening in primary care. One study [26] conducted a cost analysis, estimating $13 billion in potential savings in the USA by preventing strokes with nationwide model implementation. Other system-related findings included scalability to other EHR systems using harmonized data (n = 1) and potential applications of ML in wearable devices (n = 1). Additional system-related outcomes were not reported.

### AI models performance metrics

**Fig 4:**
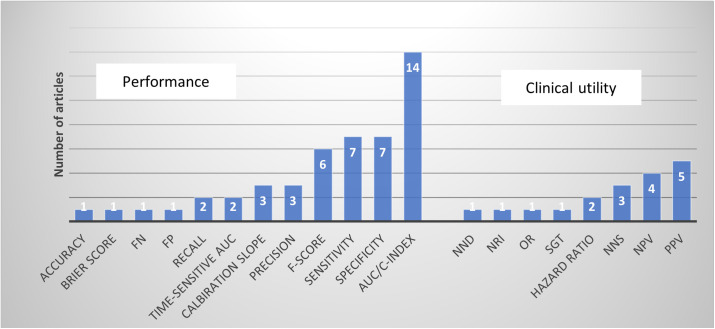
Distribution of performance metrics reported. Graph showing the variety and frequency of evaluation metrics used to assess machine learning models. Abbreviations: FN, false negatives; FP, false positives; AUC, area under the curve; C-index, concordance index; NND, number needed to detect; NRI, net reclassification improvement; OR, odds ratio; NNS, number needed to screen; NPV, negative predictive value; PPV, positive predictive value.

Performance measures were reported with considerable variability across the articles. Most studies evaluated their models’ performance using the Area Under the Curve (AUC) or Concordance Index (C-index) (n = 14, 87.5%) and the F-score (n = 7, 44%) to assess model discrimination as an indicator of performance. Fig 4 illustrates the distribution of the various metrics used across the studies.

### AI models performance comparison

Since AUC was the most frequently reported performance metric, we compared the AUC of each evaluated ML model to its respective control and summarized the results in Table 4, highlighting the highest-performing AI systems—either as standalone tools or combined with clinical tools—based on the highest reported AUC within each article. Table 4 also reports statistical significance (defined as p-value < 0.05 or non-overlapping confidence intervals) and includes performance results from models that underwent external validation. It is important to note that the performance metrics reflect results obtained within each study’s own dataset and therefore cannot be directly compared across studies. This limitation is one of the key reasons a meta-analysis was not feasible.

The AUC values for ML models ranged from 0.71 to 0.948. Eight studies (50%) demonstrated statistically significant superior performance compared to control groups, indicating effective discrimination between AF cases and non-AF cases. Among these, the random forest algorithm by [37] achieved the highest reported AUC (0.948) and F1 score (0.969). Other high-performing models included neural networks [28,36], random forest classifiers [29,35], logistic regression and naive Bayes classifiers [34] as well as natural language processing combined with other structured data [26,33]. Conversely, five studies (32%) reported no difference in performance between their ML models and control groups [22–24,30,32] indicating similar discrimination between AF and non-AF cases. However, when three of these models (19%) were combined with other clinical tools, ML methods, or structured data, a statistically significant improvement in AUC/C-index was observed compared to controls [22,24,32], except for Christopoulos et al [30] where no significant improvement was observed. The rest of studies (3 studies, 19%) did not report P values with control groups [25,27,31]; however, Shah et al [25] reported a relatively high AUC of 0.80 in external validation.

In Hill et al.[36], a UK-based prospective randomized controlled multicentric trial, the model showed no significant difference in new AF diagnoses in the full analysis population (OR = 1.15 [0.77–1.73], P = 0.486). However, in the per-protocol analysis, which includes only participants who adhered strictly to the study protocol (e.g., completed all required interventions and follow-ups as planned), the model significantly outperformed routine care (OR = 3.07 [1.57–5.81], P = 0.001). Despite COVID-19 disruptions, the trial found the ML algorithm effective in identifying high-risk patients, diagnosing AF in 5.3% of high-risk versus 0.6% of low-risk participants. The NNS was 12, much better than the NNS of 70 for opportunistic screening.

### Variables/model predictors

**Fig 5:**
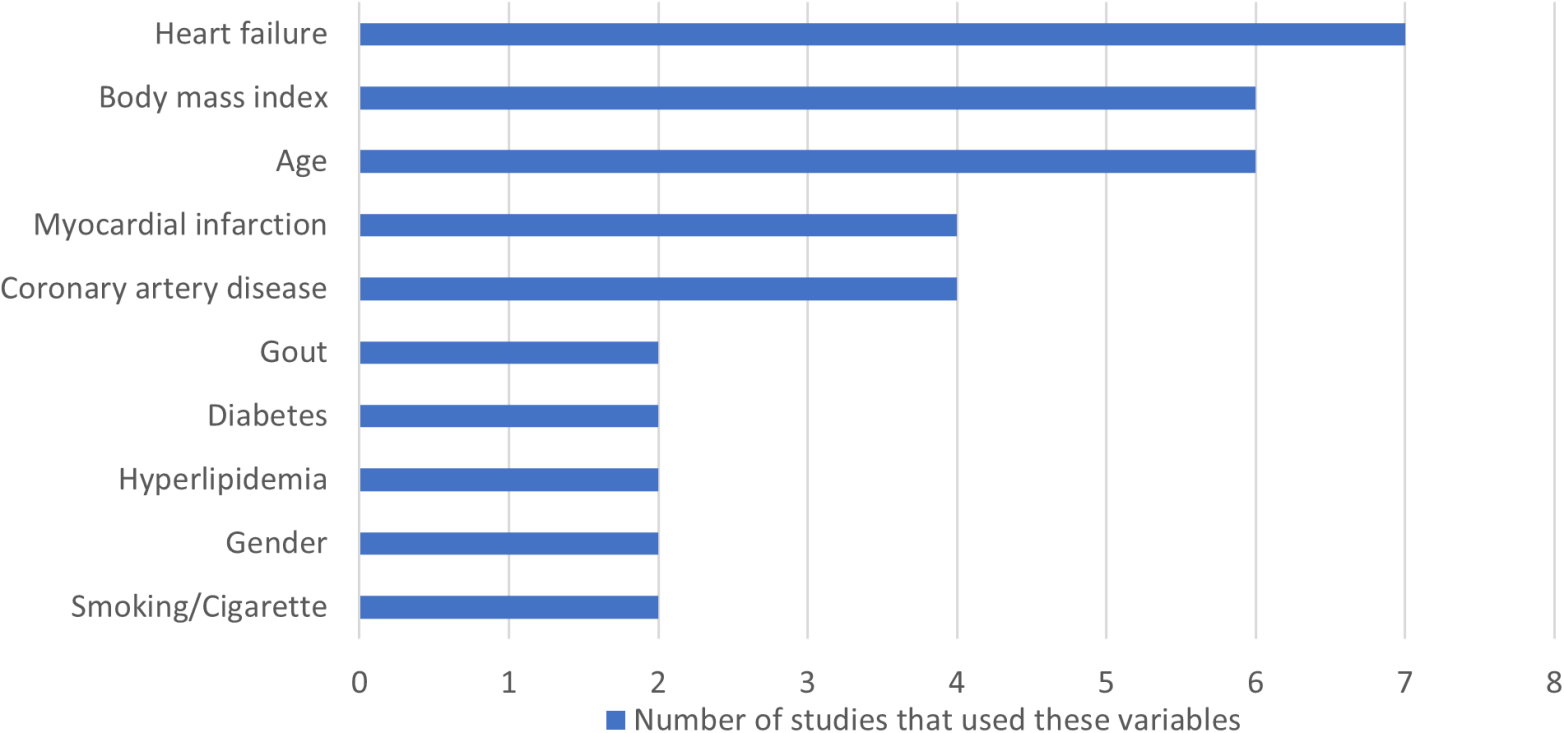
Frequently reported predictor variables. Visualization of the most common clinical and demographic variables used as predictors in the machine learning models.

S2 Table summarizes the key variables and predictors used in the ML models for AF detection in this review. These variables are categorized into demographics, clinical findings, lab/imaging features, diagnoses, procedures, and miscellaneous factors. Due to the heterogeneity in how each variable’s impact was assessed and reported across studies, direct comparison of variable strength was not feasible. Instead, we counted the frequency with which each variable appeared across studies, as shown in the supplemental material. This count represents the number of times a specific variable was included as a predictor in the ML models reviewed. To perform this count, we reviewed the methods and feature selection sections of each study and noted variables explicitly identified as predictors in their models. Fig 5 highlights variables that were reported in more than one study, suggesting their potential significance in AF detection as commonly used predictors.

In the demographics category, age, gender, and smoking emerged as the most frequently cited variables. For clinical findings, body mass index—both static measurements and changes over time—was prominent, along with signs of heart failure such as edema. Imaging-based variables frequently mentioned included anatomical metrics like left atrial diameter, right ventricular end-diastolic volume, diastolic flow, and left ventricular hypertrophy, which are all potentially linked to cardiac strain or heart failure. Other notable predictors were reduced exercise capacity and left ventricular late gadolinium enhancement, indicative of replacement fibrosis. Lower chronotropic response, abnormal blood pressure responses during exercise, and lower diastolic blood pressure at peak exercise were also associated with increased AF risk, as reported by Bhattacharya et al.[34].

Lab-based variables included hyperlipidemia and renal function tests. In the diagnoses category, cardiovascular conditions were most prevalent predictors, with heart failure as the most reported variable, notably with time-varying heart failure within the last 91 days, as reported by Hill (2019) [28]. Hypertension was frequently reported, including arterial, pulmonary, and venous forms, along with systolic and diastolic blood pressure records.

In terms of lab-based variables, hyperlipidemia and renal function tests were commonly mentioned. The diagnoses category predominantly featured cardiovascular conditions, with heart failure being the most frequently reported predictor—particularly time-varying heart failure within the last 91 days, as noted by Hill (2019) [28]. Hypertension was another frequently reported variable, encompassing arterial, pulmonary, and venous forms, along with systolic and diastolic blood pressure measurements. Myocardial infarction, coronary artery disease, and peripheral artery disease were also frequently reported variables. Other cardiovascular-related diagnoses as variables for prediction included chronic obstructive pulmonary disease (COPD), rheumatologic diseases, valvular issues (particularly mitral valve), and sleep disorders. Cardiovascular procedures such as ablation and angiography were also frequently mentioned.

Additional commonly reported variables included gout, diabetes, and various medications. Notably, diuretics (likely related to hypertension or heart failure), oral anticoagulants (non-AF related), antinausea drugs, and analgesics—including potent opioids—were also frequently mentioned as predictor variable.

### Critical appraisal

#### Assessment of bias.

**Fig 6:**
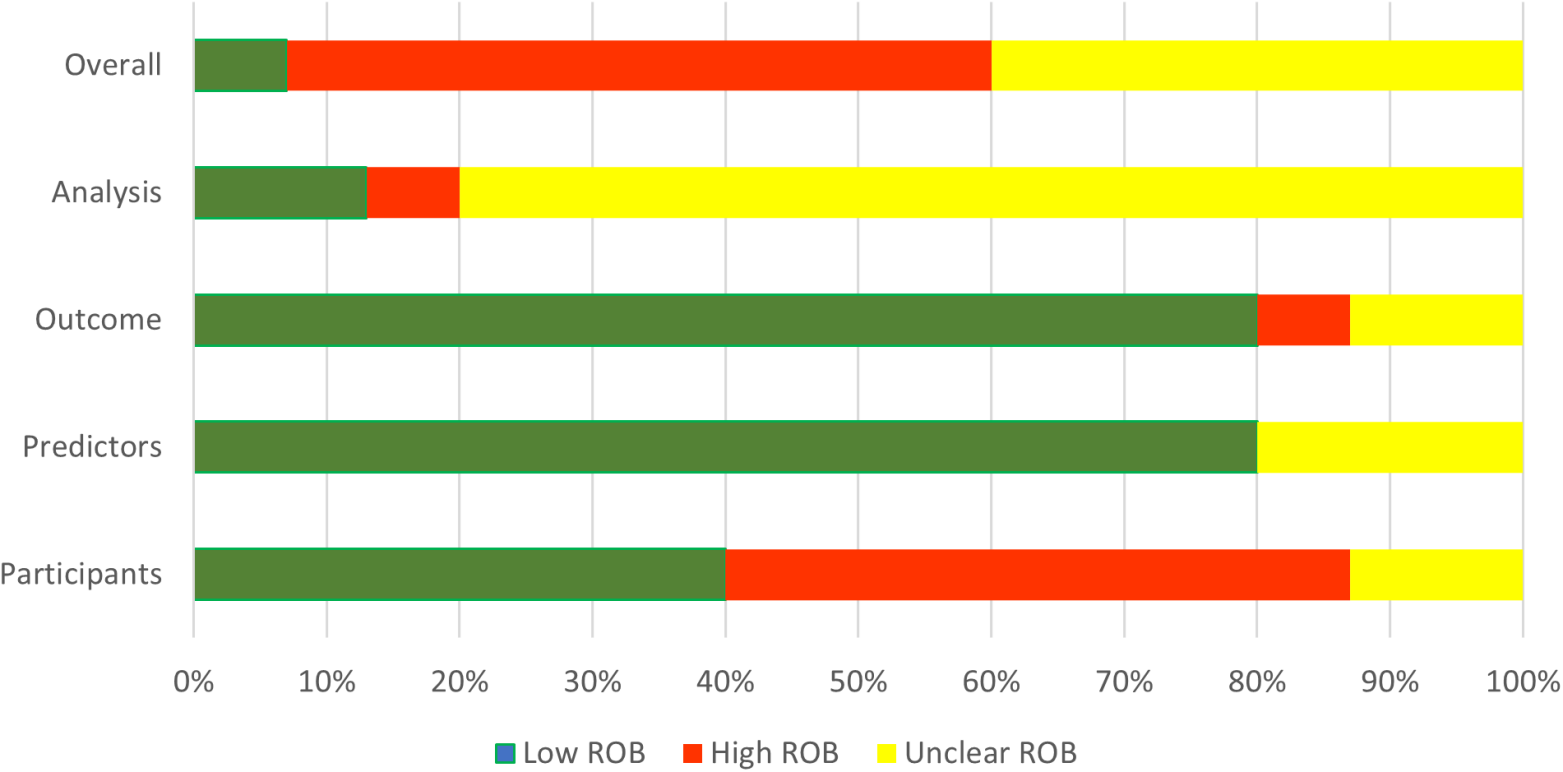
Risk of bias across studies. Summary of the risk of bias assessment for included studies using the PROBAST tool. Abbreviations: PROBAST, prediction model risk of bias assessment tool.

Out of the 16 articles included in this review, 15 were assessed using the PROBAST tool, while one clinical trial was excluded as it did not meet the criteria for PROBAST evaluation. Among the 15 studies, the overall risk of bias was high in 8 studies (53%), primarily due to issues with participant selection methods. Only one study (7%) was rated as having a low risk of bias. The remaining six studies (40%) were deemed to have an unclear risk of bias, largely due to insufficient information about how the analysis was conducted, particularly the handling of missing data. Notably, 12 studies (80%) exhibited an unclear risk of bias in the analysis domain. Additional sources of unclear risk of bias were identified in specific domains, including 2 studies (13%) with unclear risk related to participant selection, 3 studies (20%) with unclear bias in predictors, and 2 studies (13%) with unclear risk in the outcome domain. Fig 6 summarizes overall risk of bias across articles. S1 Table summarizes the individual results of the critical appraisal in each article.

#### Model validation and data processing.

S1 Table details the validation approaches and methods of feature selection used in model development. Among the 16 studies, four models underwent both internal and external validation (25%), while the remaining articles conducted only internal validation. Notably, one article did not perform any validation, as their primary aim was to compare population characteristics rather than assess performance metrics. The most common method of internal validation was a random split of training and test sets (n = 9, 56%), while the most common method for external validation was geographical splitting (n = 5, 31%). Calibration was not assessed in 12 studies (75%), and missing data were not properly handled in 8 studies (50%). The number of potential variables/features for model development varied significantly, ranging from 13 to 26,000. However, the number of final variables was relatively consistent across articles generally ranging from 13 to 19. The selection method for candidate predictors was primarily based on prior knowledge (n = 7, 44%). However, the approach for selecting final predictors was frequently unspecified in six studies (38%), with the Least Absolute Shrinkage and Selection Operator (LASSO) used in two studies (13%).

#### Applicability and reproducibility.

**Fig 7:**
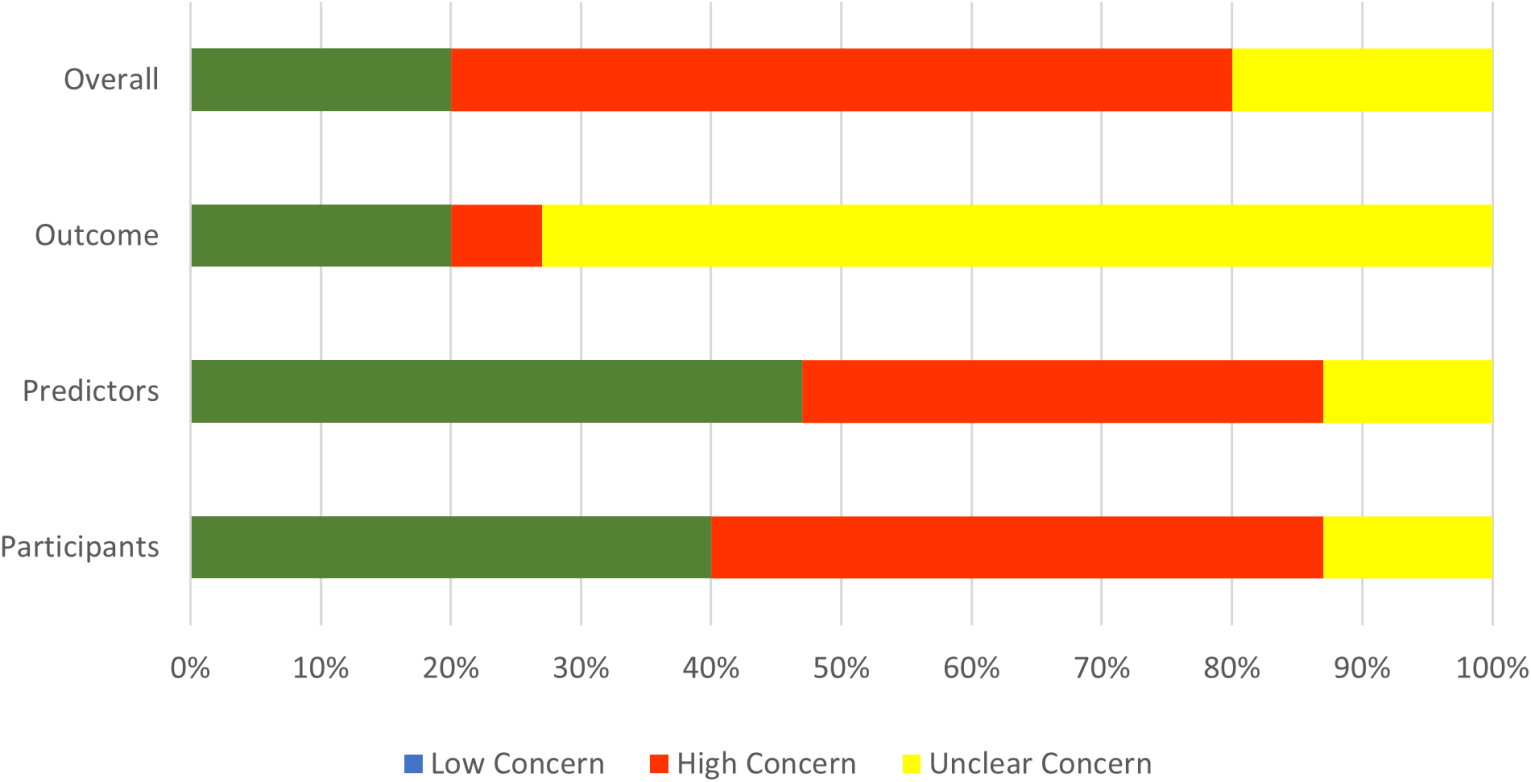
Concerns regarding applicability. Assessment of concerns related to the applicability of included studies using the PROBAST framework. Abbreviations: PROBAST, prediction model risk of bias assessment tool.

Among the 15 studies evaluated using PROBAST, the majority of studies (n = 9 studies, 60%) were rated as having high concern for overall applicability to clinical practice in primary care. These concerns were mainly due to the irrelevance of the setting and participant selection (n = 7 studies, 47%) and the lack of applicability of certain predictors in primary care (n = 6 studies, 40%). One study (7%) also showed applicability concerns due to issues with outcome definitions. Additionally, 11 studies (73%) had unclear applicability due to insufficient reporting on patient-, provider-, or system-related outcomes. Fig 7 shows overall applicability assessment.

#### MI-CLAIM checklist assessment.

Part 1 (Study Design): All the 16 studies (100%) clearly stated the research question and clinical problem. Nearly all (n = 15, 94%) described cohort characteristics, and representativeness to clinical settings reflecting a good adherence to study design standards.

Part 2 (Data and Optimization): Most studies (n = 15, 94%) detailed data origin and format, and (n = 13, 81%) provided details on the models evaluated. However, only 56%(n = 9) reported on training-test set independence, while data transformations are rarely addressed (n = 3, 19%), indicating gaps in data handling transparency.

Part 3 (Model Performance): Most articles (n = 15, 94%) presented baseline-to-model performance comparisons and 88% (n = 14) specifying algorithm performance metrics. Clinical utility metrics are noted in 69% (n = 11) with minor gaps in clarity. Overall, model performance documentation is comprehensive, particularly for baseline comparisons.

Part 4 (Percentage of Model Code Sharing): Overall, model code sharing is limited. Only 7% of studies (n = 1) fully shared their models’ codes, while most studies (n = 10, 62%) restrict access or do not share their code as shown in S1 Fig. Summary of MI-CLAIM checklist is shown in S1 Fig.

#### Cross-mapping of model effectiveness and applicability.

To visualize how often models achieved in terms of predictive performance and clinical applicability, we developed a matrix mapping these two dimensions (Table 5). The majority of studies (n = 7, 44%) fell into the moderate effectiveness/ high or unclear applicability quadrant, indicating limited readiness for clinical use, while only three models (19%) demonstrated both high effectiveness and low applicability concern. These findings underscore the need to improve the predictive performance of future models while using primary care-based metrics.

**Table 5:** Matrix mapping study-level performance against PROBAST-rated applicability: number (percentage).

		Performance		
		High	Moderate	Low
**Applicability** **(PROBAST)**	**High concern**	1 (6%)	5 (31%)	3 (19%)
	**Unclear concern**	1 (6%)	2 (12%)	0
	**Low concern**	3 (19%)	1 (6%)	0

Performance was classified as high, moderate, or low based on Area-Under-Curve and demonstrated superiority to control. High performance: AUC ≥ 0.9 and superiority to control. Moderate performance: AUC ≥ 0.9 or superiority to control (but not both). Low performance: Neither AUC ≥ 0.9 nor superiority demonstrated. Applicability tiers were derived from PROBAST.

## Discussion

We conducted a systematic review to assess the effectiveness of ML methods in detecting new cases of AF using EHR in primary care settings. Additionally, we evaluated the quality and practical applicability of these models. The key findings from our review are summarized below.

### ML methods are effective in detecting AF using EHR in primary care

For a thorough evaluation of predictive models’ performance, two key metrics—calibration and discrimination—are essential. Calibration assesses how well predicted probabilities align with observed outcomes, while discrimination, often measured by AUC or C-index, reflects a model’s ability to differentiate between high- and low-risk individuals [39]. Both metrics range from 0 to 1, with higher values (closer to 1.0) indicating better performance. C-index above 0.75 suggests strong discrimination [39]. However, for utility in clinical practice, discrimination above 0.9 is required for regulatory considerations [40,41]. In this review, 81% of studies (n = 13) reported an AUC above 0.75, demonstrating strong discrimination. However, the lack of reported calibration metrics limits a comprehensive assessment of model accuracy. A meta-analysis was not feasible due to high risk of bias in many studies and substantial heterogeneity in both performance metrics and the datasets used to develop ML models. As a result, cross-study comparisons were inappropriate. Instead, we evaluated model performance relative to control groups within each study.

When compared to the performance (discrimination) of control groups, half of the models (50%, n = 8) in this review outperformed the controls with supervised models accounting for 63% (n = 5) of these superior models. In contrast, models based on unsupervised learning methods or deep learning models showed only moderate performance. Notably, combining machine learning models (both supervised and unsupervised) with clinical tools, such as CHARGE-AF or ICD codes for AF, significantly improved model performance (discrimination). This indicates that optimal results are achieved when predictive models are used in conjunction with established clinical tools.

These findings underscore the considerable potential of ML models for AF screening in primary care. Compared to traditional models like CHADS2 (C-index 0.674) and CHARGE-AF (C-index 0.71) [7] or in head-to-head comparisons with control groups, ML models in this review demonstrated superior diagnostic performance, improving the identification of high-risk patients and contributing to better clinical outcomes. For the healthcare system, ML models in this review significantly reduced the NNS to 12, as shown in the prospective clinical trial in this review, Hill et al.’s [36], compared to 70 with conventional methods [7]. At a population level, applying such models could improve resource utilization and reduce the burden on primary care. Hill et al. [42] further demonstrated this, in another article, through cost-effectiveness analyses, showing substantial health benefits with an incremental gain of 80,669 QALYs, linked to fewer AF-related complications.

### Most current ML models have high risk of bias

Our analysis revealed a spectrum of bias risks ranging from uncertain to high across most studies, primarily driven by participant selection, model overfitting, and lack of external validation. Specifically, selection bias emerged as a significant concern in 56% (n = 9) of the included studies, often due to single-center data, non-random participant selection, or exclusion of participants with incomplete data. Notably, only 25% (n = 4) of the studies conducted external validation, a critical step in mitigating the risk of overfitting and enhancing model generalizability. This observation aligns with previous findings on the prevalence of this issue in similar research contexts [43,44]. Another common source of bias was inadequate reporting on handling missing data. Only five studies (31%) reported their approach to missing data, while three studies (19%) included only participants with complete data. This oversight is a common issue in ML predictive model development. As Nijman et al. [45] noted, most ML models developed between 2018 and 2019 lacked adequate strategies for handling missing data, which can introduce significant non-reporting bias and undermine model reliability.

### Most current ML models show limited applicability in primary care

While ML models show promise for AF screening, concerns remain regarding their applicability in primary care. Most studies used retrospective cohort designs, which can introduce selection and recall bias. Although primary care data were utilized, the inclusion of inpatient records, imaging, and laboratory results—often not readily accessible in primary care—further limits models’ practical applicability. We emphasize the need for using representative datasets to the intended clinical context when developing ML models inline with previous research. To enhance clinical relevance, ML models should be developed using datasets that accurately reflect the target care setting, as emphasized in prior research [46]. Furthermore, the predominance of North American datasets restricts generalizability to other populations.

Furthermore, important clinical metrics such as positive predictive value and number needed to screen were inconsistently reported, limiting understanding of these models’ practical relevance in primary care. Nevertheless, studies that included these metrics demonstrated notable improvements in screening efficiency as discussed above. Reproducibility also remains a challenge, as limited sharing of model codes restricts broader validation and real-world implementation.

### ML models were able to identify novel predictors for AF in primary care

Our review shows that ML models effectively identified both traditional and novel AF risk factors. Classic predictors, including age, gender, smoking, hypertension, type 2 diabetes, and myocardial infarction, were consistent with established tools like CHARGE-AF [1,47]. Unlike traditional models that rely on static numerical values, some ML models integrated dynamic predictors, enabling the identification of non-linear relationships. For example, one model in this review [28] highlighted that recent heart failure episodes (within the last three months), changes in BMI, and recent, frequent blood pressure measurements emerged as stronger predictors of AF risk compared to a single heart failure diagnosis or isolated BP readings. These dynamic variables offer potential targets for AF screening in primary care, potentially improving screening accuracy.

Beyond traditional risk factors, our review identified imaging markers linked to heart failure or diastolic dysfunction, such as left atrial diameter, left ventricular hypertrophy, and fibrosis, consistent with existing literature [48,49]. Features like exercise intolerance and reduced chronotropic response, associated with cardiovascular stress reactivity, were identified by our review as predictors of AF aligning with prior research [50]. Additionally, clinical conditions such as valvular disease, chronic inflammatory conditions (e.g., rheumatologic diseases, kidney disease), and COPD were identified by our review as predictors associated with AF, consistent with broader research [51,52].

Notably, our review found that cancer and cancer-related medications, such as antiemetics and potent opioids, emerged as significant AF predictors, AF’s increased incidence during chemotherapy. This observation aligns with existing literature, which report a peak in AF incidence within 90 days post-cancer diagnosis, likely due to inflammation and oxidative stress [53]. These findings underscore an opportunity to target high-risk cancer patients for AF screening in primary care.

Finally, our review identified gout as a novel AF risk factor in the reviewed studies. This finding aligns with external research, such as the meta-analysis by Deng et al.[54], which demonstrated that gout increases AF risk by 33%, highlighting hyperuricemia’s role in AF pathogenesis. These results suggest a new opportunity to target patients with gout for AF screening in primary care.

### Future directions

To enhance the effectiveness and applicability of ML methods in AF screening, future research should prioritize several key areas. First, consistent and transparent reporting of calibration metrics—such as calibration plots or Brier scores—is essential to determine whether predicted probabilities accurately reflect real-world outcomes. This is particularly important in clinical settings, where accurate risk estimates guide decisions about screening and treatment. Second, addressing and reporting missing data is important for improving model robustness. Third, external validation across diverse but primary care-based datasets is necessary to mitigate overfitting and improve generalizability. Fourth, prospective cohort designs are recommended to reduce selection and recall bias. Fifth, developing models that prioritize clinically accessible predictors over ECG or imaging variables, will enable seamless integration into routine primary care workflows without adding to the burden on healthcare providers. Sixth, using clinically meaningful metrics—such as positive predictive value and number needed to screen—along with standardized performance measures, will enhance the practical relevance of findings for primary care settings and facilitate cross-study comparisons. Finally, ensuring reproducibility through the sharing of code and methodologies is an important practice to replicate and test models on different contexts.

### Strengths

This review provides a comprehensive analysis of the effectiveness of EHR-based ML models for AF detection in primary care, alongside a rigorous evaluation of bias sources and applicability limitations. It offers valuable insights for primary care providers to critically assess these tools, which have the potential to surpass current standard practices, and presents actionable recommendations to address existing gaps and support their practical integration into primary care workflows.

### Limitations

This study has several limitations. Our search strategy excluded ML methods that rely on ECG, imaging, laboratory tests, and wearable devices, which may have narrowed the scope of our analysis. A meta-analysis was not possible due to substantial variations in performance metrics and follow-up durations, limiting cross-study comparisons. Furthermore, the predominance of retrospective single-center cohort designs and North American data registries also restricted the generalizability of our findings. Regarding missing data, we recorded unreported items as “not reported” or “unclear” in our extraction sheet. Due to the extent and variability of missing data, we did not impute values or contact study authors but instead chose to retain these studies and highlight the issue as a gap and a limitation in the current literature.

## Conclusions

In conclusion, this study highlights the promise of machine learning models in leveraging electronic health records for early atrial fibrillation detection in primary care. These models demonstrate strong potential for scalable and efficient screening by utilizing real-world data. However, addressing challenges such as limited generalizability, lack of external validation, and insufficient clinically relevant metrics is essential to enhance their applicability. By overcoming these barriers, these models can transform AF screening and significantly improve patient outcomes in primary care.

## Supporting information

S1 Appendix Search Strategy. (DOCX)

S1 Fig Summary of MICLAIM checklist. (DOCX)

S1 Table Data extraction (CHARMS) and risk of bias evaluations (PROBAST) for each study. (XLSX)

S2 Table Summary of atrial fibrillation predictors included across the reviewed ML models. (DOCX)

S1 Protocol Full registered protocol for the review (PROSPERO CRD42023390603). (PDF)

S1 Checklist Completed PRISMA 2020 checklist for systematic reviews. (DOCX)
