# Supplementary material for: Are Machine Learning methods effective in detecting undiagnosed atrial fibrillation in primary care settings using electronic health records? A systematic review
Source: PLOS Digit Health. 2025 Oct 14;4(10):e0001009. doi: 10.1371/journal.pdig.0001009 (PMC12520348; doi:10.1371/journal.pdig.0001009)
Supplement: S1 Appendix — (DOCX) [file pdig.0001009.s001.docx]

**S1 Appendix: Search Strategy**

Search strategies – Atrial fibrillation AND screening AND AI AND PHC versus PHC OR EHR

| Database | Platform | Date searched | # of records |
| --- | --- | --- | --- |
| MEDLINE ALL | Ovid | 2023/05/03 | 607 |
| Embase Classic+Embase | Ovid | 2023/05/03 | 1374 |
| CENTRAL | Cochrane Library | 2023/03/15 | 310 |
| CINAHL | EBSCOhost | 2023/05/03 | 286 |
| Web of Science Core Collection | Web of Science | 2023/05/03 | 672 |
| Scopus | (Scopus) | 2023/05/03 | 671 |
| IEEE Xplore | (IEEE Xplore) | 2023/05/03 | 540 (6 RIS files) |

Databases to Search

MEDLINE (Ovid)

Ovid MEDLINE(R) ALL <1946 to May 02, 2023>

1 Atrial Fibrillation/ or (atrial fibrillation* or auricular fibrillation* or atrium fibrillation* or afib or a fib).ti,ab,kf. or Atrial Flutter/ or (atrial flutter* or atrium flutter* or auricular flutter*).ti,ab,kf. 108279

2 Atrial Fibrillation/di or Atrial Flutter/di or Mass Screening/ or "Predictive Value of Tests"/ or screen*.ti,ab,kf. or Diagnosis/ or "Diagnostic Techniques and Procedures"/ or (detect* or diagnos* or identif* or test* or ((systemat* or opportunist* or target* or population or mass) adj2 assess*) or asymptomatic).ti,ab,kf. 11175819

3 artificial intelligence/ or exp machine learning/ 90105

4 computer heuristics/ or expert systems/ or fuzzy logic/ or exp knowledge bases/ or natural language processing/ or exp neural networks, computer/ or sentiment analysis/ 82866

5 (AI or ((artificial* or computat* or machine* or multitask* or "multi task*" or reinforcement or transfer or shallow* or competitive) adj1 (intelligen* or learn*)) or ((bayes* or neural or "echo state*" or "generative adversarial") adj1 (method* or network* or naive* or learning* or reservoir*)) or classifier* or ((approximate or automated or "case based" or computer*) adj1 (heuristic* or reasoning or soft or evolutionary or vision)) or ("data driven" or ((data or text) adj1 mining)) or (deep adj2 learning) or ((expert or intelligent* or multiagent* or "multi agent*") adj1 system*) or ensemble or (fuzzy adj1 (classif* or cluster* or control* or cognitive or inference or logic or rule* or system*)) or (knowledge adj1 (acquisition* or representation*)) or "natural language processing*" or "neural network*" or "prediction model*" or "predictive model*" or "support vector*" or SVM or (random adj2 forest*) or ((semisupervised or supervised or unsupervised) adj3 learning) or (feature adj (extraction or learning or selection or engineering)) or representation learning or "auto encod*" or autoencod* or generative model* or "principal component analys*" or "reservoir computing" or "echo state network*" or "case-based reasoning" or metaheuristic* or "meta heuristic*" or "soft computing" or "approximate reasoning" or "evolutionary computing" or ((bio-inspired or clustering or evolutionary or genetic or learning) adj1 algorithm*) or ((document or pattern*) adj1 classifi*) or "knowledge base*" or knowledgebase* or "knowledge engineering" or ((ambient or swarm) adj1 intelligen*) or (adaptive adj1 (boost* or system*)) or "back propagat*" or backpropagat* or "dimensionality reduction" or "k nearest neighbo*" or (decision adj (forest* or tree*)) or transformer* or anns or feed forward or perceptron* or artificial nn or artificial nns or XGBoost or "gradient boost*" or "CART model*" or "classification and regression tree*" or SMOTE or "synthetic minority oversampling technique*" or "k means cluster*" or "local linear embedding" or "independent component analys*" or ((multi layer or multilayer or deep) adj architecture*) or restricted Boltzmann machine* or ConvNet or CNN or SIANN or ResNet or U-Net or GRU or gated recurrent unit* or LSTM or long short-term memory or GANs).mp. 571996

6 3 or 4 or 5 581119

7 exp General Practice/ or nurse practitioners/ or family nurse practitioners/ or nurses, community health/ or *physicians/ or general practitioners/ or geriatricians/ or physicians, family/ or physicians, primary care/ or exp Physician Assistants/ or Community Health Workers/ or primary health care/ or (clinic? or practi* or (primary adj2 (healthcare or care)) or physician* or refer* or visit* or outpatient* or consult* or family or communit* or ambulatory or centre? or center? or office or internist* or geriatric* or gerontolog*).ti,ab,kf. 5660878

8 exp Medical Records Systems, Computerized/ or exp Health Records, Personal/ or (personal adj (health record* or medical record*)).ti,ab,kf. or ((electronic* or online or on-line or digital*) adj2 (health record* or medical record* or personal record* or patient record*)).ti,ab,kf. or ((web or internet or computer*) adj3 (health record* or medical record* or personal record* or patient record*)).ti,ab,kf. or (ehr? or phr? or ephr? or emr or paehr?).ti,ab,kf. or (patient adj2 portal*).ti,ab,kf. 95869

9 7 or 8 5705794

10 1 and 2 and 6 and 9 607

<https://proxy.library.mcgill.ca/login?url=https://ovidsp.ovid.com/ovidweb.cgi?T=JS&NEWS=N&PAGE=main&SHAREDSEARCHID=7WJoJNvMRKPwn5ZlvDyAk8G46oV70jBSLRciALX1FMkyxBPw85w0jjx9jpwYR0nDa>

Embase (Ovid)

Embase Classic+Embase <1947 to 2023 May 02>

1 atrial fibrillation/ or paroxysmal atrial fibrillation/ or persistent atrial fibrillation/ or (atrial fibrillation* or auricular fibrillation* or atrium fibrillation* or afib or a fib).ti,ab,kf. or Atrial Flutter/ or (atrial flutter* or auricular flutter*).ti,ab,kf. 211390

2 Atrial Fibrillation/di or Mass Screening/ or "Predictive Value of Tests"/ or screen*.ti,ab,kf. or Diagnosis/ or "Diagnostic Techniques and Procedures"/ or (detect* or diagnos* or identif* or test* or ((systemat* or opportunist* or target* or population or mass) adj2 assess*) or asymptomatic).ti,ab,kf. 15800209

3 exp artificial intelligence/ or big data/ or computer vision/ or "decision tree"/ or exp machine learning/ or natural language processing/ 447457

4 expert system/ or fuzzy logic/ or knowledge base/ 17664

5 (AI or ((artificial* or computat* or machine* or multitask* or "multi task*" or reinforcement or transfer or shallow* or competitive) adj1 (intelligen* or learn*)) or ((bayes* or neural or "echo state*" or "generative adversarial") adj1 (method* or network* or naive* or learning* or reservoir*)) or classifier* or ((approximate or automated or "case based" or computer*) adj1 (heuristic* or reasoning or soft or evolutionary or vision)) or ("data driven" or ((data or text) adj1 mining)) or (deep adj2 learning) or ((expert or intelligent* or multiagent* or "multi agent*") adj1 system*) or ensemble or (fuzzy adj1 (classif* or cluster* or control* or cognitive or inference or logic or rule* or system*)) or (knowledge adj1 (acquisition* or representation*)) or "natural language processing*" or "neural network*" or "prediction model*" or "predictive model*" or "support vector*" or SVM or (random adj2 forest*) or ((semisupervised or supervised or unsupervised) adj3 learning) or (feature adj (extraction or learning or selection or engineering)) or representation learning or "auto encod*" or autoencod* or generative model* or "principal component analys*" or "reservoir computing" or "echo state network*" or "case-based reasoning" or metaheuristic* or "meta heuristic*" or "soft computing" or "approximate reasoning" or "evolutionary computing" or ((bio-inspired or clustering or evolutionary or genetic or learning) adj1 algorithm*) or ((document or pattern*) adj1 classifi*) or "knowledge base*" or knowledgebase* or "knowledge engineering" or ((ambient or swarm) adj1 intelligen*) or (adaptive adj1 (boost* or system*)) or "back propagat*" or backpropagat* or "dimensionality reduction" or "k nearest neighbo*" or (decision adj (forest* or tree*)) or transformer* or anns or feed forward or perceptron* or artificial nn or artificial nns or XGBoost or "gradient boost*" or "CART model*" or "classification and regression tree*" or SMOTE or "synthetic minority oversampling technique*" or "k means cluster*" or "local linear embedding" or "independent component analys*" or ((multi layer or multilayer or deep) adj architecture*) or restricted Boltzmann machine* or ConvNet or CNN or SIANN or ResNet or U-Net or GRU or gated recurrent unit* or LSTM or long short-term memory or GANs).mp. 750836

6 3 or 4 or 5 856022

7 general practice/ or nurse practitioners/ or adult nurse practitioner/ or family nurse practitioners/ or gerontologic nurse practitioner/ or *physicians/ or general practitioner/ or gerontologist/ or internist/ or osteopathic physician/ or physician assistant/ or health auxiliary/ or exp primary health care/ or (clinic? or practi* or (primary adj2 (healthcare or care)) or physician* or refer* or visit* or outpatient* or consult* or family or communit* or ambulatory or centre? or center? or office or internist* or geriatric* or gerontolog*).ti,ab,kf. 8010872

8 exp electronic health record/ or (personal adj (health record* or medical record*)).ti,ab,kf. or ((electronic* or online or on line or digital*) adj2 (health record* or medical record* or personal record* or patient record*)).ti,ab,kf. or ((web or internet or computer*) adj3 (health record* or medical record* or personal record* or patient record*)).ti,ab,kf. or (ehr? or phr? or ephr? or emr? or paehr?).ti,ab,kf. or (patient adj2 portal*).ti,ab,kf. 144059

9 7 or 8 8066125

10 1 and 2 and 6 and 9 1374

<https://proxy.library.mcgill.ca/login?url=https://ovidsp.ovid.com/ovidweb.cgi?T=JS&NEWS=N&PAGE=main&SHAREDSEARCHID=7JhA5sPtlZLZpwfI0G2GwFR3GBLGOztON7smlNNEWWI6Fz563z8qOpYCweKcFpBof>

CENTRAL (Cochrane Library/Wiley)

Old search:

310 records

March 15, 2023

Search Name:

Date Run: 15/03/2023 17:40:28

Comment:

ID Search Hits

#1 [mh ^"Atrial Fibrillation"] OR (("atrial" NEAR/2 fibrillation*) OR ("auricular" NEAR/2 fibrillation*) OR ("atrium" NEAR/2 fibrillation*) OR afib OR "a fib"):ti,ab,kw OR [mh ^"Atrial Flutter"] OR (("atrial" NEAR/2 flutter*) OR ("atrium" NEAR/2 flutter*) OR ("auricular" NEAR/2 flutter*)):ti,ab,kw 15566

#2 MeSH descriptor: [Atrial Fibrillation] this term only and with qualifier(s): [diagnosis - DI] 977

#3 MeSH descriptor: [Atrial Flutter] this term only and with qualifier(s): [diagnosis - DI] 68

#4 [mh ^"Mass Screening"] OR [mh ^"Predictive Value of Tests"] OR screen*:ti,ab,kw OR [mh ^Diagnosis] OR [mh ^"Diagnostic Techniques and Procedures"] OR (detect* OR diagnos* OR identif* OR test* OR ((systemat* OR opportunist* OR target* OR population OR mass) NEAR/2 assess*) OR asymptomatic):ti,ab,kw 801956

#5 #2 OR #3 OR #4 801956

#6 [mh ^"artificial intelligence"] OR [mh "machine learning"] 1096

#7 [mh ^"computer heuristics"] OR [mh ^"expert systems"] OR [mh ^"fuzzy logic"] OR [mh "knowledge bases"] OR [mh ^"natural language processing"] OR [mh "neural networks, computer"] OR [mh ^"sentiment analysis"] 670

#8 (AI OR ((artificial* OR computat* OR machine* OR multitask* OR ("multi" NEAR/2 task*) OR reinforcement OR transfer OR shallow* OR competitive) NEAR/1 (intelligen* OR learn*)) OR ((bayes* OR neural OR ("echo" NEAR/2 state*) OR "generative adversarial") NEAR/1 (method* OR network* OR naive* OR learning* OR reservoir*)) OR classifier* OR ((approximate OR automated OR "case based" OR computer*) NEAR/1 (heuristic* OR reasoning OR soft OR evolutionary OR vision)) OR ("data driven" OR ((data OR text) NEAR/1 mining)) OR (deep NEAR/2 learning) OR ((expert OR intelligent* OR multiagent* OR ("multi" NEAR/2 agent*)) NEAR/1 system*) OR ensemble OR (fuzzy NEAR/1 (classif* OR cluster* OR control* OR cognitive OR inference OR logic OR rule* OR system*)) OR (knowledge NEAR/1 (acquisition* OR representation*)) OR ("natural language" NEAR/2 processing*) OR ("neural" NEAR/2 network*) OR ("prediction" NEAR/2 model*) OR ("predictive" NEAR/2 model*) OR ("support" NEAR/2 vector*) OR SVM OR (random NEAR/2 forest*) OR ((semisupervised OR supervised OR unsupervised) NEAR/3 learning) OR (feature NEXT (extraction OR learning OR selection OR engineering)) OR "representation learning" OR ("auto" NEAR/2 encod*) OR autoencod* OR ("generative" NEAR/2 model*) OR ("principal component" NEAR/2 analys*) OR "reservoir computing" OR ("echo state" NEAR/2 network*) OR "case-based reasoning" OR metaheuristic* OR ("meta" NEAR/2 heuristic*) OR "soft computing" OR "approximate reasoning" OR "evolutionary computing" OR ((bio-inspired OR clustering OR evolutionary OR genetic OR learning) NEAR/1 algorithm*) OR ((document OR pattern*) NEAR/1 classifi*) OR ("knowledge" NEAR/2 base*) OR knowledgebase* OR "knowledge engineering" OR ((ambient OR swarm) NEAR/1 intelligen*) OR (adaptive NEAR/1 (boost* OR system*)) OR ("back" NEAR/2 propagat*) OR backpropagat* OR "dimensionality reduction" OR ("k nearest" NEAR/2 neighbo*) OR (decision NEXT (forest* OR tree*)) OR transformer* OR anns OR "feed forward" OR perceptron* OR "artificial nn" OR "artificial nns" OR XGBoost OR ("gradient" NEAR/2 boost*) OR ("CART" NEAR/2 model*) OR ("classification and regression" NEAR/2 tree*) OR SMOTE OR ("synthetic minority oversampling" NEAR/2 technique*) OR ("k means" NEAR/2 cluster*) OR "local linear embedding" OR ("independent component" NEAR/2 analys*) OR (("multi layer" OR multilayer OR deep) NEXT architecture*) OR ("restricted Boltzmann" NEAR/2 machine*) OR ConvNet OR CNN OR SIANN OR ResNet OR U-Net OR GRU OR ("gated recurrent" NEAR/2 unit*) OR LSTM OR "long short-term memory" OR GANs):ti,ab,kw 47862

#9 #6 OR #7 OR #8 47874

#10 #1 AND #5 AND #9 in Trials 310

Recommended: Do not add a fourth concept, screen the records for that concept instead

CINAHL (EBSCOhost)

| # | Query | Limiters/Expanders | Last Run Via | Results |
| --- | --- | --- | --- | --- |
| S7 | S1 AND S2 AND S3 AND S6 | Expanders - Apply equivalent subjects Search modes - Boolean/Phrase | Interface - EBSCOhost Research Databases Search Screen - Advanced Search Database - CINAHL Plus with Full Text | 286 |
| S6 | S4 OR S5 | Expanders - Apply equivalent subjects Search modes - Boolean/Phrase | Interface - EBSCOhost Research Databases Search Screen - Advanced Search Database - CINAHL Plus with Full Text | 2,499,957 |
| S5 | (MH "Patient Record Systems+") OR ((TI personal OR AB personal OR SU personal) W1 ((TI "health record*" OR AB "health record*" OR SU "health record*") OR (TI "medical record*" OR AB "medical record*" OR SU "medical record*"))) OR (((TI electronic* OR AB electronic* OR SU electronic*) OR (TI online OR AB online OR SU online) OR (TI on-line OR AB on-line OR SU on-line) OR (TI digital* OR AB digital* OR SU digital*)) N2 ((TI "health record*" OR AB "health record*" OR SU "health record*") OR (TI "medical record*" OR AB "medical record*" OR SU "medical record*") OR (TI "personal record*" OR AB "personal record*" OR SU "personal record*") OR (TI "patient record*" OR AB "patient record*" OR SU "patient record*"))) OR (((TI web OR AB web OR SU web) OR (TI internet OR AB internet OR SU internet) OR (TI computer* OR AB computer* OR SU computer*)) N3 ((TI "health record*" OR AB "health record*" OR SU "health record*") OR (TI "medical record*" OR AB "medical record*" OR SU "medical record*") OR (TI "personal record*" OR AB "personal record*" OR SU "personal record*") OR (TI "patient record*" OR AB "patient record*" OR SU "patient record*"))) OR ((TI ehr# OR AB ehr# OR SU ehr#) OR (TI phr# OR AB phr# OR SU phr#) OR (TI ephr# OR AB ephr# OR SU ephr#) OR (TI emr OR AB emr OR SU emr) OR (TI paehr# OR AB paehr# OR SU paehr#)) OR ((TI patient OR AB patient OR SU patient) N2 (TI portal* OR AB portal* OR SU portal*)) | Expanders - Apply equivalent subjects Search modes - Boolean/Phrase | Interface - EBSCOhost Research Databases Search Screen - Advanced Search Database - CINAHL Plus with Full Text | 58,081 |
| S4 | (MH "Family Practice") OR (MH "Internal Medicine") OR (MH "Nurse Practitioners") OR (MH "Adult Nurse Practitioners") OR (MH "Family Nurse Practitioners") OR (MH "Gerontologic Nurse Practitioners") OR (MH "Community Health Nursing+") OR (MM "Physicians") OR (MH "Physicians, Family") OR (MH "Geriatricians") OR (MH "Physician Assistants") OR (MH "Community Health Workers") OR (MH "Preventive Health Care") OR (MH "Diagnostic Services") OR (MH "Health Screening+") OR (MH "Community Health Services") OR (MH "primary health care") OR (MH "Ambulatory Care") OR (MH "Ambulatory Care Nursing") OR ((TI clinic# OR AB clinic# OR SU clinic#) OR (TI practi* OR AB practi* OR SU practi*) OR ((TI primary OR AB primary OR SU primary) N2 ((TI healthcare OR AB healthcare OR SU healthcare) OR (TI care OR AB care OR SU care))) OR (TI physician* OR AB physician* OR SU physician*) OR (TI refer* OR AB refer* OR SU refer*) OR (TI visit* OR AB visit* OR SU visit*) OR (TI outpatient* OR AB outpatient* OR SU outpatient*) OR (TI consult* OR AB consult* OR SU consult*) OR (TI family OR AB family OR SU family) OR (TI communit* OR AB communit* OR SU communit*) OR (TI ambulatory OR AB ambulatory OR SU ambulatory) OR (TI centre# OR AB centre# OR SU centre#) OR (TI center# OR AB center# OR SU center#) OR (TI office OR AB office OR SU office) OR (TI internist* OR AB internist* OR SU internist*) OR (TI geriatric* OR AB geriatric* OR SU geriatric*) OR (TI gerontolog* OR AB gerontolog* OR SU gerontolog*)) | Expanders - Apply equivalent subjects Search modes - Boolean/Phrase | Interface - EBSCOhost Research Databases Search Screen - Advanced Search Database - CINAHL Plus with Full Text | 2,474,860 |
| S3 | (MH "Artificial Intelligence") OR (MH "Expert Systems") OR (MH "Knowbots") OR (MH "Knowledge Bases+") OR (MH "Machine Learning+") OR (MH "Natural Language Processing") OR (MH "Neural Networks (Computer)") OR (AI OR ((artificial* OR computat* OR machine* OR multitask* OR "multi task*" OR reinforcement OR transfer OR shallow* OR competitive) N1 (intelligen* OR learn*)) OR ((bayes* OR neural OR "echo state*" OR "generative adversarial") N1 (method* OR network* OR naive* OR learning* OR reservoir*)) OR classifier* OR ((approximate OR automated OR "case based" OR computer*) N1 (heuristic* OR reasoning OR soft OR evolutionary OR vision)) OR ("data driven" OR ((data OR text) N1 mining)) OR (deep N2 learning) OR ((expert OR intelligent* OR multiagent* OR "multi agent*") N1 system*) OR ensemble OR (fuzzy N1 (classif* OR cluster* OR control* OR cognitive OR inference OR logic OR rule* OR system*)) OR (knowledge N1 (acquisition* OR representation*)) OR "natural language processing*" OR "neural network*" OR "prediction model*" OR "predictive model*" OR "support vector*" OR SVM OR (random N2 forest*) OR ((semisupervised OR supervised OR unsupervised) N3 learning) OR (feature W1 (extraction OR learning OR selection OR engineering)) OR "representation learning" OR "auto encod*" OR autoencod* OR "generative model*" OR "principal component analys*" OR "reservoir computing" OR "echo state network*" OR "case-based reasoning" OR metaheuristic* OR "meta heuristic*" OR "soft computing" OR "approximate reasoning" OR "evolutionary computing" OR ((bio-inspired OR clustering OR evolutionary OR genetic OR learning) N1 algorithm*) OR ((document OR pattern*) N1 classifi*) OR "knowledge base*" OR knowledgebase* OR "knowledge engineering" OR ((ambient OR swarm) N1 intelligen*) OR (adaptive N1 (boost* OR system*)) OR "back propagat*" OR backpropagat* OR "dimensionality reduction" OR "k nearest neighbo*" OR (decision W1 (forest* OR tree*)) OR transformer* OR anns OR "feed forward" OR perceptron* OR "artificial nn" OR "artificial nns" OR XGBoost OR "gradient boost*" OR "CART model*" OR "classification and regression tree*" OR SMOTE OR "synthetic minority oversampling technique*" OR "k means cluster*" OR "local linear embedding" OR "independent component analys*" OR (("multi layer" OR multilayer OR deep) W1 architecture*) OR "restricted Boltzmann machine*" OR ConvNet OR CNN OR SIANN OR ResNet OR U-Net OR GRU OR "gated recurrent unit*" OR LSTM OR "long short-term memory" OR GANs) | Expanders - Apply equivalent subjects Search modes - Boolean/Phrase | Interface - EBSCOhost Research Databases Search Screen - Advanced Search Database - CINAHL Plus with Full Text | 150,647 |
| S2 | (MH "Atrial Fibrillation/DI") OR (MH "Atrial Flutter/DI") OR (MH "Diagnosis") OR (MH "Diagnosis, Cardiovascular+") OR (MH "Diagnosis, Computer Assisted") OR (MH "Diagnostic Imaging") OR (MH "Early Diagnosis") OR (MH "Health Screening") OR (MH "Predictive Value of Tests") OR (TI screen* OR AB screen* OR SU screen*) OR ((TI detect* OR AB detect* OR SU detect*) OR (TI diagnos* OR AB diagnos* OR SU diagnos*) OR (TI identif* OR AB identif* OR SU identif*) OR (TI test* OR AB test* OR SU test*) OR (((TI systemat* OR AB systemat* OR SU systemat*) OR (TI opportunist* OR AB opportunist* OR SU opportunist*) OR (TI target* OR AB target* OR SU target*) OR (TI population OR AB population OR SU population) OR (TI mass OR AB mass OR SU mass)) N2 (TI assess* OR AB assess* OR SU assess*)) OR (TI asymptomatic OR AB asymptomatic OR SU asymptomatic)) | Expanders - Apply equivalent subjects Search modes - Boolean/Phrase | Interface - EBSCOhost Research Databases Search Screen - Advanced Search Database - CINAHL Plus with Full Text | 2,889,067 |
| S1 | (MH "Atrial Fibrillation") OR ((TI "atrial fibrillation*" OR AB "atrial fibrillation*" OR SU "atrial fibrillation*") OR (TI "auricular fibrillation*" OR AB "auricular fibrillation*" OR SU "auricular fibrillation*") OR (TI "atrium fibrillation*" OR AB "atrium fibrillation*" OR SU "atrium fibrillation*") OR (TI afib OR AB afib OR SU afib) OR (TI "a fib" OR AB "a fib" OR SU "a fib")) OR (MH "Atrial Flutter") OR ((TI "atrial flutter*" OR AB "atrial flutter*" OR SU "atrial flutter*") OR (TI "atrium flutter*" OR AB "atrium flutter*" OR SU "atrium flutter*") OR (TI "auricular flutter*" OR AB "auricular flutter*" OR SU "auricular flutter*")) | Expanders - Apply equivalent subjects Search modes - Boolean/Phrase | Interface - EBSCOhost Research Databases Search Screen - Advanced Search Database - CINAHL Plus with Full Text | 43,286 |

Web of Science Core Collection (Web of Science): A&HCI , BKCI-SSH , BKCI-S , CCR-EXPANDED , ESCI , IC , CPCI-SSH , CPCI-S , SCI-EXPANDED , SSCI

# Web of Science Search Strategy (v0.1)

# Database: Web of Science Core Collection

# Entitlements:

- WOS.IC: 1993 to 2023

- WOS.CCR: 1985 to 2023

- WOS.SCI: 1900 to 2023

- WOS.AHCI: 1975 to 2023

- WOS.BHCI: 2005 to 2023

- WOS.BSCI: 2005 to 2023

- WOS.ESCI: 2005 to 2023

- WOS.ISTP: 1990 to 2023

- WOS.SSCI: 1900 to 2023

- WOS.ISSHP: 1990 to 2023

# Searches:

Search:

TS=(AI OR ((artificial* OR computat* OR machine* OR multitask* OR "multi task*" OR reinforcement OR transfer OR shallow* OR competitive) NEAR/1 (intelligen* OR learn*)) OR ((bayes* OR neural OR "echo state*" OR "generative adversarial") NEAR/1 (method* OR network* OR naive* OR learning* OR reservoir*)) OR classifier* OR ((approximate OR automated OR "case based" OR computer*) NEAR/1 (heuristic* OR reasoning OR soft OR evolutionary OR vision)) OR ("data driven" OR ((data OR text) NEAR/1 mining)) OR (deep NEAR/2 learning) OR ((expert OR intelligent* OR multiagent* OR "multi agent*") NEAR/1 system*) OR ensemble OR (fuzzy NEAR/1 (classif* OR cluster* OR control* OR cognitive OR inference OR logic OR rule* OR system*)) OR (knowledge NEAR/1 (acquisition* OR representation*)) OR "natural language processing*" OR "neural network*" OR "prediction model*" OR "predictive model*" OR "support vector*" OR SVM OR (random NEAR/2 forest*) OR ((semisupervised OR supervised OR unsupervised) NEAR/3 learning) OR (feature NEAR/0 (extraction OR learning OR selection OR engineering)) OR "representation learning" OR "auto encod*" OR autoencod* OR "generative model*" OR "principal component analys*" OR "reservoir computing" OR "echo state network*" OR "case-based reasoning" OR metaheuristic* OR "meta heuristic*" OR "soft computing" OR "approximate reasoning" OR "evolutionary computing" OR ((bio-inspired OR clustering OR evolutionary OR genetic OR learning) NEAR/1 algorithm*) OR ((document OR pattern*) NEAR/1 classifi*) OR "knowledge base*" OR knowledgebase* OR "knowledge engineering" OR ((ambient OR swarm) NEAR/1 intelligen*) OR (adaptive NEAR/1 (boost* OR system*)) OR "back propagat*" OR backpropagat* OR "dimensionality reduction" OR "k nearest neighbo*" OR (decision NEAR/0 (forest* OR tree*)) OR transformer* OR anns OR "feed forward" OR perceptron* OR "artificial nn" OR "artificial nns" OR XGBoost OR "gradient boost*" OR "CART model*" OR "classification and regression tree*" OR SMOTE OR "synthetic minority oversampling technique*" OR "k means cluster*" OR "local linear embedding" OR "independent component analys*" OR (("multi layer" OR multilayer OR deep) NEAR/0 architecture*) OR "restricted Boltzmann machine*" OR ConvNet OR CNN OR SIANN OR ResNet OR U-Net OR GRU OR "gated recurrent unit*" OR LSTM OR "long short-term memory" OR GANs)

AND

TS=(screen* OR detect* OR diagnos* OR identif* OR test* OR ((systemat* OR opportunist* OR target* OR population OR mass) NEAR/2 assess*) OR asymptomatic)

AND

TS=("atrial fibrillation*" OR "auricular fibrillation*" OR "atrium fibrillation*" OR afib OR "a fib" OR "atrial flutter*" OR "atrium flutter*" OR "auricular flutter*")

AND

(

TS=("clinic" OR "clinics" OR practice OR practitioner* OR (primary NEAR/2 (healthcare OR care)) OR physician* OR refer* OR visit* OR outpatient* OR consult* OR (family NEAR/2 (doctor* OR medicine)) OR communit* OR ambulatory OR centre* OR center* OR office OR internist* OR geriatric* OR gerontolog*)

OR

TS=( ( personal NEAR/1 ( "health record*" OR "medical record*" ) ) OR ( ( electronic* OR online OR on-line OR digital* ) NEAR/2 ( "health record*" OR "medical record*" OR "personal record*" OR "patient record*" ) ) OR ( ( web OR internet OR computer* ) NEAR/3 ( "health record*" OR "medical record*" OR "personal record*" OR "patient record*" ) ) OR ehr OR ehrs OR phr OR phrs OR ephr OR ephrs OR emr OR emrs OR paehr OR paehrs OR ( patient NEAR/2 portal* ) )

)

Date Run: Wed May 03 2023 19:34:39 GMT-0400 (Eastern Daylight Time)

Results: 672

<https://www.webofscience.com/wos/woscc/summary/f34d8f2d-0ccc-4998-8f70-1bb66893ace3-81c34ae1/relevance/1>

Scopus

671 records on May 3, 2023

Advanced Search:

TITLE-ABS("atrial fibrillation*" OR "auricular fibrillation*" OR "atrium fibrillation*" OR afib OR "a fib" OR "atrial flutter*" OR "atrium flutter*" OR "auricular flutter*")

AND

TITLE-ABS(screen* OR detect* OR diagnos* OR identif* OR test* OR ((systemat* OR opportunist* OR target* OR population OR mass) W/2 assess*) OR asymptomatic)

AND

TITLE-ABS-KEY(AI OR ((artificial* OR computat* OR machine* OR multitask* OR "multi task*" OR reinforcement OR transfer OR shallow* OR competitive) W/1 (intelligen* OR learn*)) OR ((bayes* OR neural OR "echo state*" OR "generative adversarial") W/1 (method* OR network* OR naive* OR learning* OR reservoir*)) OR classifier* OR ((approximate OR automated OR "case based" OR computer*) W/1 (heuristic* OR reasoning OR soft OR evolutionary OR vision)) OR ("data driven" OR ((data OR text) W/1 mining)) OR (deep W/2 learning) OR ((expert OR intelligent* OR multiagent* OR "multi agent*") W/1 system*) OR ensemble OR (fuzzy W/1 (classif* OR cluster* OR control* OR cognitive OR inference OR logic OR rule* OR system*)) OR (knowledge W/1 (acquisition* OR representation*)) OR "natural language processing*" OR "neural network*" OR "prediction model*" OR "predictive model*" OR "support vector*" OR SVM OR (random W/2 forest*) OR ((semisupervised OR supervised OR unsupervised) W/3 learning) OR (feature W/1 (extraction OR learning OR selection OR engineering)) OR "representation learning" OR "auto encod*" OR autoencod* OR "generative model*" OR "principal component analys*" OR "reservoir computing" OR "echo state network*" OR "case-based reasoning" OR metaheuristic* OR "meta heuristic*" OR "soft computing" OR "approximate reasoning" OR "evolutionary computing" OR ((bio-inspired OR clustering OR evolutionary OR genetic OR learning) W/1 algorithm*) OR ((document OR pattern*) W/1 classifi*) OR "knowledge base*" OR knowledgebase* OR "knowledge engineering" OR ((ambient OR swarm) W/1 intelligen*) OR (adaptive W/1 (boost* OR system*)) OR "back propagat*" OR backpropagat* OR "dimensionality reduction" OR "k nearest neighbo*" OR (decision W/1 (forest* OR tree*)) OR transformer* OR anns OR "feed forward" OR perceptron* OR "artificial nn" OR "artificial nns" OR XGBoost OR "gradient boost*" OR "CART model*" OR "classification and regression tree*" OR SMOTE OR "synthetic minority oversampling technique*" OR "k means cluster*" OR "local linear embedding" OR "independent component analys*" OR (("multi layer" OR multilayer OR deep) W/1 architecture*) OR "restricted Boltzmann machine*" OR ConvNet OR CNN OR SIANN OR ResNet OR U-Net OR GRU OR "gated recurrent unit*" OR LSTM OR "long short-term memory" OR GANs)

AND

(

TITLE-ABS({clinic} OR {clinics} OR practice OR practitioner* OR (primary W/2 (healthcare OR care)) OR physician* OR refer* OR visit* OR outpatient* OR consult* OR (family W/2 (doctor* OR medicine)) OR communit* OR ambulatory OR centre* OR center* OR office OR internist* OR geriatric* OR gerontolog*)

OR

TITLE-ABS ( ( personal W/1 ( "health record*" OR "medical record*" ) ) OR ( ( electronic* OR online OR on-line OR digital* ) W/2 ( "health record*" OR "medical record*" OR "personal record*" OR "patient record*" ) ) OR ( ( web OR internet OR computer* ) W/3 ( "health record*" OR "medical record*" OR "personal record*" OR "patient record*" ) ) OR ehr OR ehrs OR phr OR phrs OR ephr OR ephrs OR emr OR emrs OR paehr OR paehrs OR ( patient W/2 portal* ) )

)

IEEE Xplore

540 records on May 3, 2023

Advanced Search:

All Metadata:

"atrial fibrillation" OR "auricular fibrillation" OR "atrium fibrillation" OR afib OR "a fib"

AND

All Metadata:

screen* OR detect* OR diagnos* OR identif* OR test* OR asymptomatic

All Metadata:

clinic OR clinics OR practice OR practitioners OR primary OR physicians OR refer* OR visit* OR outpatients OR consult* OR family OR community OR ambulatory OR office OR internists OR geriatric* OR gerontolog* OR records OR EHR OR EHRS OR portals

Did not include: centre* OR center* OR phr OR phrs OR ephr OR ephrs OR emr OR emrs OR paehr OR
