# Supplementary material for: Are Machine Learning methods effective in detecting undiagnosed atrial fibrillation in primary care settings using electronic health records? A systematic review
Source: PLOS Digit Health. 2025 Oct 14;4(10):e0001009. doi: 10.1371/journal.pdig.0001009 (PMC12520348; doi:10.1371/journal.pdig.0001009)
Supplement: S1 Fig — (DOCX) [file pdig.0001009.s002.docx]

**S1 Fig: Summary of MICLAIM checklist**
