# Supplementary material for: Are Machine Learning methods effective in detecting undiagnosed atrial fibrillation in primary care settings using electronic health records? A systematic review
Source: PLOS Digit Health. 2025 Oct 14;4(10):e0001009. doi: 10.1371/journal.pdig.0001009 (PMC12520348; doi:10.1371/journal.pdig.0001009)
Supplement: S2 Table — (DOCX) [file pdig.0001009.s004.docx]

|  | S2 Table: Summary of atrial fibrillation predictors included across the reviewed ML models | | | | | |  |  |
| --- | --- | --- | --- | --- | --- | --- | --- | --- |
|  | **Demographics** | **Vital sings** | **Diagnosis/Procedure** | **Signs/Symptoms** | **Lab** | **Imaging** | **Medications** | **Miscellaneous** |
| Elkin 2021 |  |  | Diagnosis of Atrial Fibrillation  Otherwise not individually reported |  |  |  |  |  |
| Mukherjee 2019 |  |  | non-negated mention of AF in the clinical text |  |  |  |  |  |
| Karnik 2012 |  |  | Pulmonary hypertension, venous hypertension, | Negative peripheral edema | Magnesium measurement, Plasma glucose measurement | X-ray of chest: pleural effusion, Air trapping | Lasix, Diuretics | Cigarette |
| Hill 2019 | Body mass index (3Q prior index), Body mass index (4Q prior index), Body mass index (2Q prior index), Body mass index (1Q prior index), Weight (1Q prior index), Change in BMI (2Q to 1Q prior index), Weight (3Q prior index), | Systolic blood pressure measurement count (1Q prior index) | Heart failure (1Q prior index), Coronary heart disease (1Q prior index), Myocardial infarction (2Q prior index), Coronary heart disease (2Q prior index), Heart failure (3Q prior index), Heart failure (4Q prior index), Heart failure (2Q prior index), Myocardial infarction (3Q prior index), Body mass index (1Q prior index), Coronary heart disease (3Q prior index), Myocardial infarction (1Q prior index) |  |  | Left ventricular hypertrophy (1Q prior index) |  | Current smoker |
| Tiwari 2020 |  |  | Collection of venous blood by venipuncture, Office or other outpatient or emergency visit assessment for an established patient, Office or other outpatient for a new patient, Essential hypertension, Immunization, Therapeutic, prophylactic, or diagnostic injection, Cough, Abdominal pain, Intravenous infusion, hydration; each additional hour, Hyperlipidemia | History of clinical finding in subject, General examination of patient, |  |  | Ondansetron, Sodium Chloride, Acetaminophen /Hydrocodone Bitartrate, Fentanyl, Acetaminophen / Oxycodone, Calcium Chloride /Potassium Chloride / Sodium Chloride / Sodium Lactate, Injectable Solution, Naloxone Hydrochloride, Injectable Solution, Oxycodone Hydrochloride, Ondansetron Disintegrating Oral Tablet, |  |
| Shah 2020 |  |  |  |  |  |  |  | upto 1000 features/100 stop wods |
| Christopoulos 2020 |  |  | ECG features from leads (I,II, V1-6) |  |  |  |  |  |
| Sekelj 2021 |  |  |  |  |  |  |  | Refer to Hill 2019 |
| Khurshid 2022 |  |  | ECG features from leads (I,II, V1-6) |  |  |  |  |  |
|  | **Demographics** | **Vital sings** | **Diagnosis/Procedure** | **Signs/Symptoms** | **Lab** | **Imaging** | **Medications** | **Miscellaneous** |
| Ashburner 2022 |  |  | Obesity, Mitral valve disorder, Mitral valve insufficiency, Mitral valve stenosis, Supraventricular tachycardia, Premature atrial contractions, Myocardial infarctions, Chronic kidney disease, Chronic kidney disease: severe, Other cardiomyopathy, Congestive heart failure, Coronary artery disease, Peripheral vascular disease |  |  |  |  |  |
| Bhattacharya 2021 | Age (+) | Percentage of max heart rate achieved at peak exercise (-), Heart rate recovery at 1 min post-exercise (-), abnormal blood pressure response during exercise test (+), Diastolic blood pressure at peak exercise (-) |  | Heart rate at peak stress (-), Exercise metabolic equivalent (-), Exercise time (-), New York Heart Association (+), |  | Left atrial diameter (+), Septal myectomy (+), LV late gadolinium enhancement on CMR (+), ratio of early diastolic mitral flow velocity to the late diastolic mitral flow velocity; (-), ratio of early diastolic mitral flow velocity to the early diastolic mitral septal annulus, LV global longitudinal early diastolic strain rate (-) motion velocity (+), LV global longitudinal, peak systolic strain rate (+), Dyspnea on exertion (+), | Diuretic treatment (+) |  |
| Sung 2022 | Age, female, body mass index | heart rate, pulse pressure | diabetes', 'cardiovascular', 'electrocardiogram', | 'numbness'', | Triglyceride, Platelet count, creatinine, blood urea nitrogen |  |  | unit', 'day', 'middle', 'heart', 'family', 'visit', 'ago' |
| Dykstra 2022 | Age, weight | Diastolic blood pressure, Systolic blood pressure | Referral for angiography, COPD, Congenital heart disease, |  |  | LA volume, RV EDV, LV EDV, LV mass, LVEF, RV ESV, Significant VHD, LV ESV, LV cardiac output | Antiarrythemic use (non-AF related), Oral anticoagulant use (non-AF related), Diuretic use |  |
| Hill 2022 |  |  |  |  |  |  |  | Refer to Hill 2019 |
| Ramesh 2022 | Age, ethnicity, Sex, |  | Heart failure, Non-atrial fibirillation electrophysiology procedure, Rheumatic valve disease, Other mitral valve disease, COPD, Gout, Chronic ischemic heart disease |  |  |  |  |  |
| Hu 2019 | Age in years, Age groups, Gender |  | Follow up for endpoint in years, Hypertension, COPD, Rheumatologic disease, Hyperlipidemia, Diabetes, CVA/TIA, Heart failure, Sleep disorder, Cancer, Vascular disease, Gout |  |  |  |  | "Mean CHA2DS2-VASc score (SD)" |
| Table 3: Key variables and predictors used in the ML models. Karnick used terms and not real numbers per each variable. (positive correlation) (+), (negative correlation) (-). AF: Atrial fibrillation. ECG: Electrocardiogram. 1Q refers to one-quarter of the year = 91 days. Specific doses reported in Tiwari 2022: 2 ML Ondansetron 2 MG/ML Injection, 1000 ML Sodium Chloride 9 MG/ML Injection, Acetaminophen 325 MG / Hydrocodone Bitartrate 5 MG Oral Tablet, 2 ML Fentanyl 0.05 MG/ML Injection, Acetaminophen 325 MG / Oxycodone Hydrochloride 5 MG Oral Tablet, Calcium Chloride 0.0014 MEQ/ML /Potassium Chloride 0.004 MEQ/ML / Sodium Chloride 0.103 MEQ/ML / Sodium Lactate 0.028 MEQ/ML Injectable Solution, Naloxone Hydrochloride 0.4 MG/ML Injectable Solution, Oxycodone Hydrochloride 5 MG Oral Tablet, Ondansetron 4 MG Disintegrating Oral Tablet. | | | | | | | | |

| Variable as reported in sheet 3 | Count |
| --- | --- |
| Heart failure | 7 |
| Myocardial infarction | 4 |
| Coronary artery disease | 4 |
| Body mass index | 6 |
| Age | 6 |
| COPD | 3 |
| Gout | 2 |
| Diabetes | 2 |
| Hyperlipidemia | 2 |
| Gender | 2 |
| Numbness | 1 |
| Pulse pressure | 1 |
| Cardiovascular | 1 |
| Electrocardiogram | 1 |
| Diagnosis of Atrial Fibrillation | 1 |
| Triglyceride | 1 |
| Platelet count | 1 |
| Creatinine | 1 |
| Blood urea nitrogen | 1 |
| Unit | 1 |
| Day | 1 |
| Heart rate | 1 |
| Dyspnea on exertion (+) | 1 |
| Female | 1 |
| Diuretic treatment (+) | 1 |
| Heart | 1 |
| LV global longitudinal peak systolic strain rate (+) | 1 |
| Motion velocity (+) | 1 |
| LV global longitudinal early diastolic strain rate (-) | 1 |
| Ratio of early diastolic mitral flow velocity to the early diastolic mitral septal annulus | 1 |
| Ratio of early diastolic mitral flow velocity to the late diastolic mitral flow velocity (-) | 1 |
| LV late gadolinium enhancement on CMR (+) | 1 |
| Septal myectomy (+) | 1 |
| Left atrial diameter (+) | 1 |
| New York Heart Association (+) | 1 |
| Exercise time (-) | 1 |
| Exercise metabolic equivalent (-) | 1 |
| Heart rate at peak stress (-) | 1 |
| Middle | 1 |
| Ago | 1 |
| Family | 1 |
| Antiarrhythmic use (non-AF related) | 1 |
| Vascular disease | 1 |
| Cancer | 1 |
| Sleep disorder | 1 |
| CVA/TIA | 1 |
| Rheumatologic disease | 1 |
| Hypertension | 1 |
| Follow up for endpoint in years | 1 |
| Chronic ischemic heart disease | 1 |
| Other mitral valve disease | 1 |
| Rheumatic valve disease | 1 |
| Non-atrial fibrillation electrophysiology procedure | 1 |
| Diuretic use | 1 |
| Oral anticoagulant use (non-AF related) | 1 |
| LV cardiac output | 1 |
| Visit | 1 |
| LV ESV | 1 |
| Significant VHD | 1 |
| RV ESV | 1 |
| LVEF | 1 |
| LV mass | 1 |
| LV EDV | 1 |
| RV EDV | 1 |
| LA volume | 1 |
| Congenital heart disease | 1 |
| Referral for angiography | 1 |
| Systolic blood pressure | 1 |
| Diastolic blood pressure | 1 |
| Weight | 1 |
| Abnormal blood pressure response during exercise test (+) | 1 |
| Diastolic blood pressure at peak exercise (-) | 1 |
| Percentage of max heart rate achieved at peak exercise (-) | 1 |
| Heart rate recovery at 1 min post-exercise (-) | 1 |
| Weight (3Q prior index) | 1 |
| Systolic blood pressure measurement count (1Q prior index) | 1 |
| Office or other outpatient or emergency visit assessment for an established patient | 1 |
| Left ventricular hypertrophy (1Q prior index) | 1 |
| Current smoker | 1 |
| Change in BMI (2Q to 1Q prior index) | 1 |
| Weight (1Q prior index) | 1 |
| Cigarette | 1 |
| Diuretics | 1 |
| Lasix | 1 |
| Air trapping | 1 |
| X-ray of chest: pleural effusion | 1 |
| Plasma glucose measurement | 1 |
| Magnesium measurement | 1 |
| Negative peripheral edema | 1 |
| Venous hypertension | 1 |
| Pulmonary hypertension | 1 |
| Non-negated mention of AF in the clinical text | 1 |
| Collection of venous blood by venipuncture | 1 |
| Office or other outpatient for a new patient | 1 |
| Otherwise not individually reported | 1 |
| Premature atrial contractions | 1 |
| Obesity | 1 |
| Mitral valve disorder | 1 |
| Mitral valve insufficiency | 1 |
| Mitral valve stenosis | 1 |
| Supraventricular tachycardia | 1 |
| Essential hypertension | 1 |
| Chronic kidney disease | 1 |
| Chronic kidney disease: severe | 1 |
| Other cardiomyopathy | 1 |
| Congestive heart failure | 1 |
| Peripheral vascular disease | 1 |
| Ondansetron Disintegrating Oral Tablet | 1 |
| Oxycodone Hydrochloride | 1 |
| Naloxone Hydrochloride, Injectable Solution | 1 |
| Injectable Solution | 1 |
| Calcium Chloride / Potassium Chloride / Sodium Chloride / Sodium Lactate | 1 |
| Acetaminophen / Oxycodone | 1 |
| Fentanyl | 1 |
| Acetaminophen / Hydrocodone Bitartrate | 1 |
| Sodium Chloride | 1 |
| Ondansetron | 1 |
| General examination of patient | 1 |
| History of clinical finding in subject | 1 |
| Intravenous infusion, hydration; each additional hour | 1 |
| Abdominal pain | 1 |
| Cough | 1 |
| Therapeutic, prophylactic, or diagnostic injection | 1 |
| Immunization | 1 |
| Mean CHA2DS2-VASc score (SD) | 1 |
