# Supplementary material for: Are Machine Learning methods effective in detecting undiagnosed atrial fibrillation in primary care settings using electronic health records? A systematic review
Source: PLOS Digit Health. 2025 Oct 14;4(10):e0001009. doi: 10.1371/journal.pdig.0001009 (PMC12520348; doi:10.1371/journal.pdig.0001009)
Supplement: S1 Protocol — (PDF) [file pdig.0001009.s005.pdf]

# Systematic review

A list of fields that can be edited in an update can be found here

## 1. * Review title.

Give the title of the review in English

Evaluating the effectiveness of AI for atrial fibrillation screening: A systematic review

## 2. Original language title.

For reviews in languages other than English, give the title in the original language. This will be displayed with the English language title.

## 3. * Anticipated or actual start date.

Give the date the systematic review started or is expected to start.

20/12/2022

## 4. * Anticipated completion date.

Give the date by which the review is expected to be completed.

30/09/2023

## 5. * Stage of review at time of this submission.

**This field uses answers to initial screening questions. It cannot be edited until after registration.**

Tick the boxes to show which review tasks have been started and which have been completed.

Update this field each time any amendments are made to a published record.

The review has not yet started: Yes

| Review stage | Started | Completed |
|---|---|---|
| Preliminary searches | No | No |
| Piloting of the study selection process | No | No |
| Formal screening of search results against eligibility criteria | No | No |
| Data extraction | No | No |
| Risk of bias (quality) assessment | No | No |
| Data analysis | No | No |

Provide any other relevant information about the stage of the review here.

## 6. * Named contact.

The named contact is the guarantor for the accuracy of the information in the register record. This may be any member of the review team.

Samira Abbasgholizadeh-Rahimi

## Email salutation (e.g. "Dr Smith" or "Joanne") for correspondence:

Dr Rahimi

## 7. * Named contact email.

Give the electronic email address of the named contact.

Samira.rahimi@mcgill.ca

## 8. Named contact address

Give the full institutional/organisational postal address for the named contact.

Room 301, 5858 Ch. de la Côte-des-Neiges, Montréal, QC H3S 1Z1

## 9. Named contact phone number.

Give the telephone number for the named contact, including international dialling code.

+1 514-399-9218

## 10. * Organisational affiliation of the review.

Full title of the organisational affiliations for this review and website address if available. This field may be completed as 'None' if the review is not affiliated to any organisation.

McGill University

## Organisation web address:

https://www.mcgill.ca/familymed/

https://rahimislab.ca/

## 11. * Review team members and their organisational affiliations.

Give the personal details and the organisational affiliations of each member of the review team. Affiliation refers to groups or organisations to which review team members belong. **NOTE: email and country now MUST be entered for each person, unless you are amending a published record. PLEASE USE AN INSTITUTIONAL EMAIL ADDRESS IF POSSIBLE.**

Dr Mhd Diaa Chalati. McGill University
Dr Samira Abbasgholizadeh-Rahimi. McGill University
Dr Chetan Shirvankar. McGill University

## 12. * Funding sources/sponsors.

Details of the individuals, organizations, groups, companies or other legal entities who have funded or sponsored the review.

The Canadian Institute for Health Research project grant

## Grant number(s)

State the funder, grant or award number and the date of award

## 13. * Conflicts of interest.

List actual or perceived conflicts of interest (financial or academic).

None

## 14. Collaborators.

Give the name and affiliation of any individuals or organisations who are working on the review but who are not listed as review team members. **NOTE: email and country must be completed for each person, unless you are amending a published record.**

Dr Bertrand Lebouche. McGill University
Amir Razaghizad. McGill University

## 15. * Review question.

State the review question(s) clearly and precisely. It may be appropriate to break very broad questions down into a series of related more specific questions. Questions may be framed or refined using PI(E)COS or

similar where relevant.

1. How has artificial intelligence been used in Afib screening? What are the different AI models used in Afib screening and how were they validated?

2. How effectively are artificial intelligence algorithms detecting Afib in primary care settings using EHR, preventing Afib complications (strokes, heart failure, morbidity/mortality rates), and reducing medical costs?

3. What are the limitations of AI implementation in daily practice? What are the missing knowledge gaps and future research needs?

## 16. * Searches.

State the sources that will be searched (e.g. Medline). Give the search dates, and any restrictions (e.g. language or publication date). Do NOT enter the full search strategy (it may be provided as a link or attachment below.)

We will conduct a comprehensive search of electronic databases from the date of inception to February 2023 using a search strategy developed with the assistance of a librarian (to check with the librarian for the databases). The search strategy will be applied to Ovid-MEDLINE and translated to other databases (Ovid-MEDLINE, Embase, CINAHL, Cochrane CENTRAL, Web of Science, IEEE Xplore, Scopus) after validation and revision with the team.

## 17. URL to search strategy.

Upload a file with your search strategy, or an example of a search strategy for a specific database, (including the keywords) in pdf or word format. In doing so you are consenting to the file being made publicly accessible. Or provide a URL or link to the strategy. Do NOT provide links to your search **results**.

Alternatively, upload your search strategy to CRD in pdf format. Please note that by doing so you are consenting to the file being made publicly accessible.

Do not make this file publicly available until the review is complete

## 18. * Condition or domain being studied.

Give a short description of the disease, condition or healthcare domain being studied in your systematic review.

Atrial fibrillation (Afib) is a common arrhythmia that affects a significant number of Canadians. Untreated Afib can lead to strokes and heart failure resulting in high morbidity and mortality rates in addition to rising medical costs. Current screening methods often overlook high-risk patients due to a lack of systematic

screening and fail to identify patients with silent Afib. Previous research has shown that AI algorithms can outperform current scores in Afib detection and identify new risk factors. AI methods have the potential to improve Afib detection by analyzing a large number of clinical factors using deep learning and can be applied to multiple data sets from different populations to create more reliable models. Also, AI models can easily be implemented on a population level.

To evaluate the effectiveness of AI in Afib screening, we will examine the current gap in clinical practice, review past applications of AI in Afib detection, and assess the potential for future use of AI in this area.

## 19. * Participants/population.

Specify the participants or populations being studied in the review. The preferred format includes details of both inclusion and exclusion criteria.

Inclusion: the general population (patients and healthcare professionals): men and women 18 years old

Exclusion: Patients 18 years old

## 20. * Intervention(s), exposure(s).

Give full and clear descriptions or definitions of the interventions or the exposures to be reviewed. The preferred format includes details of both inclusion and exclusion criteria.

Inclusion: Articles whose primary objective is to describe AI methods/applications in Afib screening in primary care settings using electronic health records. Articles that incorporate ECG as a part of primary care screening. Studies on AI methods in Afib screening including but not restricted to deep neural networks and machine learning methods such as LASSO, random forests, and support vector machines.

Exclusion: Articles that describe the use of AI in detecting Afib from ECG in emergency rooms, inpatient settings, or when ECG is not used for primary prevention will be excluded. Articles that do not have the primary objective of examining the use of AI in screening in primary care settings or when AI is used in data analysis only.

## 21. * Comparator(s)/control.

Where relevant, give details of the alternatives against which the intervention/exposure will be compared (e.g. another intervention or a non-exposed control group). The preferred format includes details of both inclusion and exclusion criteria.

No restrictions on comparators/control

## 22. * Types of study to be included.

Give details of the study designs (e.g. RCT) that are eligible for inclusion in the review. The preferred format

includes both inclusion and exclusion criteria. If there are no restrictions on the types of study, this should be stated.

In this review, we will include randomized and non-randomized clinical trials, case studies, case-control studies, pilot studies, case series, comparison studies, and validation studies. Additionally, we will also include review articles that have previously been conducted in this field, such as systematic reviews, scoping reviews, critical reviews, and literature reviews.

We will exclude: letters, book chapters, editorials and non-experimental studies.

## 23. Context.

Give summary details of the setting or other relevant characteristics, which help define the inclusion or exclusion criteria.

No limitation on the context of studies.

## 24. * Main outcome(s).

Give the pre-specified main (most important) outcomes of the review, including details of how the outcome is defined and measured and when these measurement are made, if these are part of the review inclusion criteria.

The main outcome of this review is the effectiveness of AI in screening patients for Afib.

### Measures of effect

Please specify the effect measure(s) for you main outcome(s) e.g. relative risks, odds ratios, risk difference, and/or 'number needed to treat.

The effectiveness of AI will be measured using the different metrics such as sensitivity, specificity, positive predictive value, accuracy, receiver operating characteristic area under the curve (ROC-AUC), precision-recall area under the curve (PRC-AUC) and F1 score.

## 25. * Additional outcome(s).

List the pre-specified additional outcomes of the review, with a similar level of detail to that required for main outcomes. Where there are no additional outcomes please state ‘None’ or ‘Not applicable’ as appropriate to the review

Secondary outcomes of interest include:

- Patient-related outcomes such as quality of life, the reduction of Afib-related complications such as strokes, heart failure and morbidity/mortality rates.

- User-related outcomes such as workload burden, the convenience of operations

- System-related outcomes such as reduction in cost.

### Measures of effect

Please specify the effect measure(s) for you additional outcome(s) e.g. relative risks, odds ratios, risk difference, and/or 'number needed to treat.

## 26. * Data extraction (selection and coding).

Describe how studies will be selected for inclusion. State what data will be extracted or obtained. State how this will be done and recorded.

Study selection

- Reviewers: Two reviewers will conduct the search strategy, select studies and extract data. Each reviewer will be blinded to the other reviewer's decision.

- Procedure: Studies will be arranged chronologically. Each reviewer will independently screen the studies.

- Disagreement Resolution: A third reviewer will be consulted to resolve the issue.

- Software: The selection process will be managed using software such as Rayyan/Endnote.

Data extraction

- Reviewers: Two reviewers will extract data independently. Each reviewer will be blinded to the other reviewer's decision.

- Disagreement Resolution: A third reviewer will be consulted to resolve the issue.

- Missing information: The authors will be contacted.

- Extracted data: Study design, population characteristics, type of AI model, characteristics of the used AI method such as accuracy and effectiveness, main results, limitations, and any other relevant information that is essential for the objective of the review will be extracted from the selected studies.

## 27. * Risk of bias (quality) assessment.

State which characteristics of the studies will be assessed and/or any formal risk of bias/quality assessment tools that will be used.

One reviewer will critically evaluate the articles using the PROBAST tool and Modified IJMEDI, while another will verify their assessment.

## 28. * Strategy for data synthesis.

Describe the methods you plan to use to synthesise data. This **must not be generic text** but should be **specific to your review** and describe how the proposed approach will be applied to your data. If meta-

analysis is planned, describe the models to be used, methods to explore statistical heterogeneity, and software package to be used.

We will follow the PRISMA-DTA guidelines to describe the search strategy, inclusion criteria, data extraction, and quality assessment. The synthesized knowledge will be narrative. The synthesis process will be accomplished by

1) Summarizing the measures of effectiveness in the studies such as sensitivity, specificity, positive predictive value, accuracy, receiver operating characteristic area under the curve (ROC-AUC), precision-recall area under the curve (PRC-AUC) and F1 score).

2) Exploring the reasons for differences among studies for example heterogeneity of study designs, methodology, population, intervention, control groups, outcomes.

3) Assessment of bias will be reported in accordance with PROBAST criteria.

4) A meta-analysis will be performed on both primary and secondary outcomes if 3 statistics are available for a single model. This can be done on the model discriminative accuracy.

## 29. * Analysis of subgroups or subsets.

State any planned investigation of ‘subgroups’. Be clear and specific about which type of study or participant will be included in each group or covariate investigated. State the planned analytic approach.

Subgroup analysis:

- Exploring the reasons for differences among studies for example heterogeneity of study designs, methodology, population, intervention, control groups, and outcomes.

- If possible, we will investigate potential effect modifiers such as age, gender, type of AI model and/or clinical setting.

## 30. * Type and method of review.

Select the type of review, review method and health area from the lists below.

### Type of review

Cost effectiveness
No

Diagnostic
No

Epidemiologic
No

Individual patient data (IPD) meta-analysis
No

Intervention

Yes

Living systematic review

No

Meta-analysis

Yes

Methodology

No

Narrative synthesis

No

Network meta-analysis

No

Pre-clinical

No

Prevention

Yes

Prognostic

No

Prospective meta-analysis (PMA)

No

Review of reviews

No

Service delivery

No

Synthesis of qualitative studies

No

Systematic review

Yes

Other

No

## Health area of the review

Alcohol/substance misuse/abuse

No

Blood and immune system

No

Cancer

No

Cardiovascular

Yes

Care of the elderly

Yes

Child health

No

Complementary therapies

No

COVID-19

No

Crime and justice

No

Dental

No

Digestive system

No

Ear, nose and throat

No

Education

No

Endocrine and metabolic disorders

No

Eye disorders

No

General interest

No

Genetics

No

Health inequalities/health equity

No

Infections and infestations

No

International development

No

Mental health and behavioural conditions

No

Musculoskeletal

No

Neurological

No

Nursing

No

Obstetrics and gynaecology

No

Oral health

No

Palliative care

No

Perioperative care

No

Physiotherapy

No

Pregnancy and childbirth

No

Public health (including social determinants of health)

No

Rehabilitation

No

Respiratory disorders

No

Service delivery

No

Skin disorders

No

Social care

No

Surgery

No

Tropical Medicine

No

Urological

No

Wounds, injuries and accidents

No

Violence and abuse

No

## 31. Language.

Select each language individually to add it to the list below, use the bin icon  to remove any added in error.

English

There is not an English language summary

## 32. * Country.

Select the country in which the review is being carried out. For multi-national collaborations select all the countries involved.

Canada

## 33. Other registration details.

Name any other organisation where the systematic review title or protocol is registered (e.g. Campbell, or The Joanna Briggs Institute) together with any unique identification number assigned by them. If extracted data will be stored and made available through a repository such as the Systematic Review Data Repository (SRDR), details and a link should be included here. If none, leave blank.

## 34. Reference and/or URL for published protocol.

If the protocol for this review is published provide details (authors, title and journal details, preferably in Vancouver format)

Add web link to the published protocol.

Or, upload your published protocol here in pdf format. Note that the upload will be publicly accessible.

No I do not make this file publicly available until the review is complete

Please note that the information required in the PROSPERO registration form must be completed in full even if access to a protocol is given.

## 35. Dissemination plans.

Do you intend to publish the review on completion?

Yes

Give brief details of plans for communicating review findings.?

## 36. Keywords.

Give words or phrases that best describe the review. Separate keywords with a semicolon or new line. Keywords help PROSPERO users find your review (keywords do not appear in the public record but are included in searches). Be as specific and precise as possible. Avoid acronyms and abbreviations unless these are in wide use.

Atrial fibrillation screening

Artificial intelligence

Machine learning

Effectiveness

Systematic review

Cardiovascular medicine

Preventive cardiology

## 37. Details of any existing review of the same topic by the same authors.

If you are registering an update of an existing review give details of the earlier versions and include a full bibliographic reference, if available.

## 38. * Current review status.

Update review status when the review is completed and when it is published.New registrations must be ongoing so this field is not editable for initial submission.

Please provide anticipated publication date

Review_Ongoing

## 39. Any additional information.

Provide any other information relevant to the registration of this review.

## 40. Details of final report/publication(s) or preprints if available.

Leave empty until publication details are available OR you have a link to a preprint (NOTE: this field is not editable for initial submission). List authors, title and journal details preferably in Vancouver format.

Give the link to the published review or preprint.
